# Characterization of the 26S proteasome network in *Plasmodium falciparum*

**DOI:** 10.1038/srep17818

**Published:** 2015-12-07

**Authors:** Lihui Wang, Claire Delahunty, Karin Fritz-Wolf, Stefan Rahlfs, Judith Helena Prieto, John R. Yates, Katja Becker

**Affiliations:** 1 Biochemistry and Molecular Biology, Interdisciplinary Research Center, Justus Liebig University, Giessen, Germany; 2 Department of Chemical Physiology, The Scripps Research Institute, La Jolla, California; 3 Max-Planck Institute for Medical Research, Heidelberg, Germany; 4 Department of Chemistry, Western Connecticut State University, Danbury, Connecticut

## Abstract

In eukaryotic cells, the ubiquitin-proteasome system as a key regulator of protein quality control is an excellent drug target. We therefore aimed to analyze the 26S proteasome complex in the malaria parasite *Plasmodium falciparum*, which still threatens almost half of the world’s population. First, we established an affinity purification protocol allowing for the isolation of functional 26S proteasome complexes from the parasite. Subunit composition of the proteasome and component stoichiometry were studied and physiologic interacting partners were identified via *in situ* protein crosslinking. Furthermore, intrinsic ubiquitin receptors of the plasmodial proteasome were determined and their roles in proteasomal substrate recognition were analyzed. Notably, PfUSP14 was characterized as a proteasome-associated deubiquitinase resulting in the concept that targeting proteasomal deubiquitinating activity in *P. falciparum* may represent a promising antimalarial strategy. The data provide insights into a profound network orchestrated by the plasmodial proteasome and identified novel drug target candidates in the ubiquitin-proteasome system.

Malaria, caused by protozoan parasites belonging to the genus *Plasmodium*, remains one of the most devastating infectious diseases. *Plasmodium falciparum* is the deadliest form and causes most malaria mortality. While clinical treatments of malaria mostly rely on antimalarial chemotherapy, the emergence of drug resistance to many currently used antimalarial drugs has highlighted an urgent need to discover novel antimalarial agents^1^,^2^. There is no doubt that advancing our understanding of cellular processes in malaria parasites will provide new targets for screening novel and effective antimalarial strategies.

In eukaryotes, protein turnover by the ubiquitin-proteasome system (UPS) is the principle mechanism by which most intracellular proteins are kept in quality check, degraded, and recycled^3^. The UPS is critical to eukaryotic cells as it governs protein homeostasis that influences various cellular processes including cell cycle, transcriptional regulation, cellular stress response, signal transduction, and cellular trafficking. The ubiquitin proteasome pathway (UPP) typically involves a reversible protein posttranslational modification called ubiquitination that covalently attaches ubiquitin to the proteins destined to be degraded^4^. In most cases, substrate proteins are first conjugated to a polyubiquitin chain (with at least four ubiquitin molecules) and subsequently recognized and degraded by the 26S proteasome, which is the major machinery for protein deconstruction^3^. The 26S proteasome is a barrel-shaped proteinase complex composed of at least 32 subunits that can be divided into a 20S core particle (CP) and a 19S regulatory particle (RP) that stacks to the 20S^5^. While the 20S executes proteolysis via peptidylglutamyl-peptide hydrolytic (PGPH) (caspase-like), trypsin-like and chymotrypsin-like proteolytic activities found in three β-subunits (β1, β2, and β5, respectively), the 19S is mainly responsible for recognition, deubiquitination, unfolding, and translocation of substrates^6^. Particularly, the 19S utilizes two different intrinsic ubiquitin receptor domains to recognize polyubiquitinylated substrates, *i.e.* the ubiquitin-interacting motif (UIM) in the Rpn10 subunit and the pleckstrin-like receptor for ubiquitin (Pru) domain in the Rpn13 subunit^7^,^8^. The 26S proteasome is a highly dynamic complex that coordinates a network encompassing many other proteins known as proteasome-interacting proteins (PIPs) to facilitate its function^9^,^10^. Notably, ubiquitin-binding proteins such as Rad23 and Dsk2 bind to the 19S via a ubiquitin-like (UBL) domain and associate polyubiquitinylated proteins via a ubiquitin-associated (UBA) domain, thus functioning as proteasome substrate shuttle factors^11^. Moreover, the 19S associates two deubiquitinases (DUBs) USP14/Ubp6 and UCH37^12^, and a ubiquitin ligase Hul5^10^, which concertedly act on editing ubiquitin chains of proteasomal substrates on site.

Protein degradation mediated by the 26S proteasome is a vitally important means of protein regulation for many cellular processes in eukaryotes^13^,^14^. Given the facts that timely protein regulation is critical for the rapid transformations of malaria parasites and that the parasites adapt to environmental stresses (*e.g.* oxidative and temperature stresses) during their life cycle progression in humans and vectors, it is rational to speculate that the 26S proteasome is essential for the survival and virulence of malaria parasites^15^. Indeed, an increasing body of research indicates that the proteasome is vital for parasite development throughout all life stages^16^,^17^,^18^,^19^, underscoring the plasmodial proteasome as a highly promising antimalarial target. However, while the 26S proteasome in mammals and yeast have been extensively studied, the 26S proteasome in pathogenic parasites including *Plasmodium* remains poorly characterized.

In this study, we explored the substrate recognition mechanism, componential integrity and functionality of the *P. falciparum* 26S proteasome. The plasmodial 26S proteasome was successfully isolated via a novel affinity-based purification method. By doing so, we unraveled for the first time the componential composition of the plasmodial 26S proteasome and shed light on a possible proteasome network in *P. falciparum*. Finally, we characterized a specific PIP as a proteasome-associated DUB in *P. falciparum*.

## Results

### Identification of intrinsic ubiquitin receptors of the *P. falciparum* 26S proteasome

**Figure 1:**
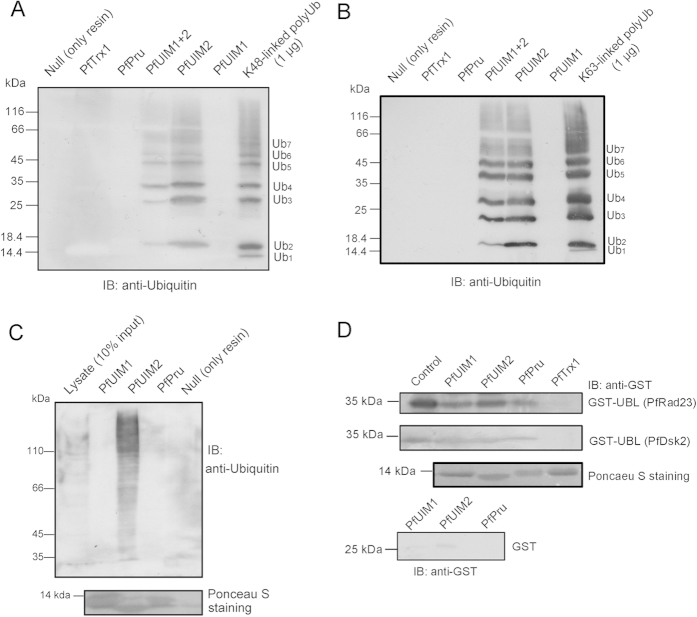
Identification of ubiquitin receptor domains in *P. falciparum* 26S proteasome components. (**A**,**B**) Assessment of the capability of PfUIM and PfPru domains in binding K48-linked (**A**) or K63-linked (**B**) polyubiquitin in a Ni-NTA pull-down assay. The His-tagged PfUIM domains, PfPru domain, and PfTrx1 (as a control) of equal molar concentrations (4.5 μM) were individually incubated with 5 μg K48-linked or K63-linked polyubiquitin of chain length ranging from one to seven (Ub_1–7_) in a Ni-NTA pull-down assay. Ubiquitin was probed by immunoblotting using an anti-ubiquitin antibody. (**C**) Association of His-tagged receptor domains with endogenous ubiquitinated proteins was examined by a Ni-NTA pull-down assay using *P. falciparum* extracts. The pulled-down ubiquitin-conjugates were detected by immunoblotting using an anti-ubiquitin antibody. The blotting membrane was stained with Ponceau S to show the amount of bait proteins. (**D**) Binding PfUIM and PfPru domains to UBL domains of PfRad23 and PfDsk2. The GST-fused UBL domains of PfRad23 and PfDsk2 were pulled down by the respective PfUIM and PfPru domains but not by PfTrx1 (as a bait control), as detected by immunoblotting using an anti-GST antibody. The blotting membrane was stained by Ponceau S to show the amount of bait proteins. GST itself is hardly pulled down by PfUIM and PfPru domains. IB, immunoblotting. Ub, ubiquitin.

To explore the elements in the *P. falciparum* 26S proteasome used for substrate recognition, *in silico* analysis of intrinsic ubiquitin receptor domains in plasmodial proteasome subunits was carried out. As a result, two putative *P. falciparum* UIM (PfUIM) domains and a putative *P. falciparum* Pru (PfPru) domain were identified in plasmodial proteasome subunits Rpn10 (PfRpn10, PF08_0109) and Rpn13 (PfRpn13, PF14_0138), respectively (Figs. S1 and S2). To assess the involvement of the identified ubiquitin receptor domains in recognition of ubiquitinylated substrates, their ubiquitin-binding capabilities were examined. The PfUIM domains, the PfPru domain, and a domain containing both PfUIMs (PfUIM1+2) were expressed and purified as hexahistidine-tagged recombinant proteins. We first tested their ubiquitin chain-binding properties including linkage type and chain length preferences in a nickel-nitrilotriacetic acid (Ni-NTA)-aided pull-down assay, in which equal molar amounts of the respective domains were incubated with K48- or K63-linked polyubiquitin chains. Intriguingly, we found that PfUIM2 and PfUIM1+2 efficiently pulled down both K48 and K63-linked polyubiquitin chains, whereas PfUIM1 and PfPru domains did not (Fig. 1A,B). Moreover, PfUIM2 and PfUIM1+2 were found to prefer longer ubiquitin chains than unconjugated ubiquitin, which is consistent with previous observations for human UIMs^20^. Next, the ability of the identified domains to serve as receptors for ubiquitinylated substrates was assessed by a Ni-NTA pull-down assay using *P. falciparum* extracts. Consistently, we found that a wide range of ubiquitin conjugates in *P. falciparum* were readily pulled down by PfUIM2 but not by PfUIM1 or PfPru domains (Fig. 1C). Our results suggest that the PfUIM2 domain may play a major role in direct recognition of ubiquitinylated substrates by the plasmodial proteasome.

It is known that UIM and Pru domains in some species interact with the ubiquitin-like (UBL) domains of proteasome substrate shuttle factors Rad23 and Dsk2, by which mechanism the proteasome receives substrates delivered by the shuttle factors^8^,^21^. The *P. falciparum* genome encodes a Rad23 homolog (PfRad23, PF10_0114) and a Dsk2 homolog (PfDsk2, PF11_0142). Both contain an N-terminal UBL domain and UBA domain(s) (Figs. S3 and S4). The UBL domains of PfRad23 and PfDsk2 were heterologously overexpressed as glutathione *S*-transferase (GST) fusion proteins, and their interactions with the His-tagged ubiquitin receptor domains were assessed in a Ni-NTA pull-down assay. We found that all PfUIM and PfPru domains can bind the GST-fused UBL domains originated from PfRad23 and PfDsk2, whereas an equimolar amount of hexahistidyl-tagged *P. falciparum* thioredoxin 1 (PfTrx1) as a bait control failed to bind the UBL domains. In a control experiment, GST itself was hardly pulled down by PfUIM and PfPru domains, confirming the specific interaction between the ubiquitin receptor domains with the UBL domains (Fig. 1D). Considering the conserved role of shuttle factors in transportation of ubiquitinylated substrates to the proteasome in eukaryotes^11^, our results suggest that the PfUIM and PfPru domains may be involved in indirect substrate recognition by the plasmodial proteasome.

### Affinity-based purification of *P. falciparum* 26S proteasome complexes

**Figure 2:**
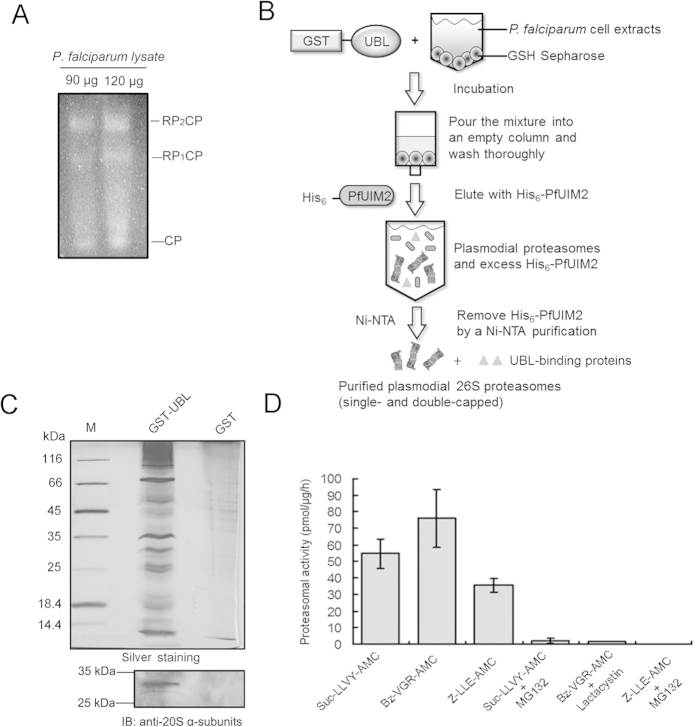
Analyses of 26S proteasome complexes of *P. falciparum*. (**A**) Native gel electrophoresis of *P. falciparum* extracts followed by an in-gel proteasome activity assay with chymotrypsin-like activity substrate Suc-LLVY-AMC. CP, 20S proteasomes; RP_1_CP, singly 19S-capped 20S proteasomes; RP_2_CP, doubly 19S-capped 20S proteasomes. (**B**) Workflow of the affinity purification of *P. falciparum* 26S proteasomes using a GST-UBL-based strategy. See Experimental procedures for more details. (**C**) Proteins in affinity-purified proteasome samples were resolved via 12% SDS-PAGE followed by silver staining analysis. A control sample from a GST-based purification was analyzed in parallel. The presence of the 20S subunits in the purified proteasome sample was confirmed by immunoblotting with an antibody (MCP231) recognizing plasmodial 20S α-subunits. (**D**) The specific chymotrypsin-like, trypsin-like, and PGPH activities of the affinity-purified *P. falciparum* proteasomes in the absence or presence of MG132 and lactacystin (10 μM) were individually determined by using the fluorogenic substrates Suc-LLVY-AMC, Bz-VGR-AMC, and Z-LLE-AMC, respectively. Each value is a mean ± S.D. from at least three independent purifications. No proteasomal activity was observed in the samples obtained from the GST-based purification.

To further analyze proteasome complexes in *P. falciparum*, we first resolved proteasome subcomplexes by using native gel electrophoresis of *P. falciparum* extracts followed by an in-gel proteasome activity assay using the fluorogenic substrate Suc-LLVY-AMC^22^. Analysis of gels showed the presence of both doubly and singly 19S-capped 20S proteasomes (*i.e.* 19S-20S-19S and 19S-20S) as well as free 20S proteasomes in the parasites, indicating that the pattern of proteasome subpopulations in *P. falciparum* is similar to that of other eukaryotic organisms^6^ (Fig. 2A).

Next, we sought to isolate 26S proteasome complexes from *P. falciparum* using an affinity purification strategy based on the GST-fused PfRad23 UBL domain (GST-UBL). In the purification, the immobilized GST-UBL binds the plasmodial proteasomes presumably via its interaction with PfUIM and PfPru domains at the 19S RP (Fig. 1D). The proteasome complexes were then eluted with hexahistidyl-tagged PfUIM2 (His_6_-PfUIM2) domain, which competes with the proteasome in binding GST-UBL, and excess His_6_-PfUIM2 could be further removed with Ni-NTA agarose (Fig. 2B). As shown in a silver-stained gel, a number of proteins were enriched via GST-UBL-based purification but not via GST-based mock purification (Fig. 2C). The presence of plasmodial proteasomes in the final eluate was detected via immunoblotting analysis. Furthermore, using a fluorogenic peptide cleavage assay we determined the specific chymotrypsin-like, trypsin-like, and post-glutamyl-peptide hydrolytic (PGPH) activities in samples of the purified plasmodial proteasomes to be 54.6 ± 8.8 pmol/μg/h, 76.08 ± 17.5 pmol/μg/h, and 35.62 ± 3.9 pmol/μg/h, respectively, which are in line with the reported values for human proteasomes isolated in a similar method^23^. Additionally, the respective proteolytic activities were shown to be inhibited by the proteasome inhibitors MG132 and lactacystin (Fig. 2D). Taken together, our results indicate that the affinity purification approach can efficiently isolate *P. falciparum* 26S proteasomes in active forms.

### Characterization of proteasome components in *P. falciparum* via proteomic analysis

We further characterized subunit composition of the *P. falciparum* 26S proteasome (Table S1) and explored potential PIPs using an integrated proteomic approach (Table S2). To stabilize assembly of proteasome complexes and freeze both stable and transient interactors with the proteasome, *P. falciparum* was subjected to *in situ* formaldehyde (1%) crosslinking prior to lysis. Proteasome complexes in the parasites were then isolated from the formaldehyde-treated or untreated parasites via GST-UBL-based affinity purification. In the end, we analyzed the affinity-purified plasmodial proteasomes from four independent purifications using formaldehyde-treated parasites, one purification using untreated parasites, and two control samples from GST-based mock purifications by using multi-dimensional protein identification technology (MudPIT). Proteins for which at least two different peptides were identified in the control were considered to be abundantly present and were thus subtracted from the list of identified proteins (see Table S3). As shown in Table S1, 32 proteasomal components corresponding to all 19S and 20S subunits were reproducibly identified in the samples from the crosslinked parasites, strongly indicating that the *P. falciparum* 26S proteasome preserves componential integrity. The relatively low recovery of 20S subunits in the purification without crosslinking indicates that the association of 19S and 20S is dynamic in the parasites (Table S1). A similar phenomenon has been observed in affinity purification of proteasomes from other organisms^24^. Moreover, no human proteasome subunits were detected.

**Figure 3:**
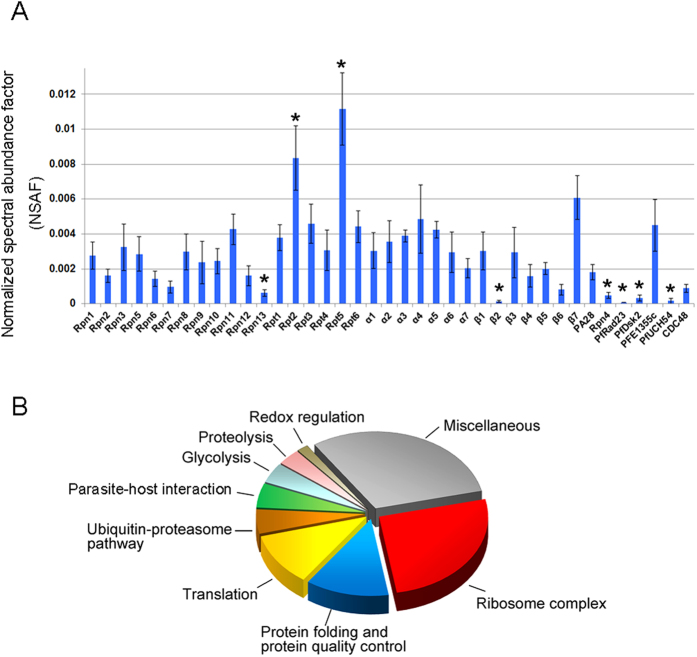
Proteomic analysis of affinity-purified plasmodial proteasomes after formaldehyde induced cross-linking. (**A**) Relative quantification of abundance of *P. falciparum* 26S proteasome components based on NSAF. Each column represents the average NSAF value of an identified protein from four independent proteasome purifications, and the error bar represents the SD of the data. Identified proteins with a statistically different (*P* < 0.01) NSAF value were marked by asterisks. None of these components were detected in the negative control samples (please see Table S3). (**B**) Classification of co-identified proteins according to their annotated roles in biological processes in *P. falciparum*.

To assess the compositional stoichiometry of plasmodial proteasome subunits, we analyzed the relative abundance of proteasome subunits by using a label-free quantitative approach based on the normalized spectral abundance factor (NSAF), which is particularly compatible with shotgun proteomics^25^. As shown in Fig. 3A, most proteasome components were found present with similar abundance, except two ATPase base subunits (Rpt2 and Rpt5) with higher abundance and two subunits (Rpn13 and β2) with low abundance. In accordance, low recovery of subunit Rpn13 has been reported in affinity purifications of human proteasomes^23^,^24^.

### Identification of potential PIPs of the *P. falciparum* 26S proteasome

It is well acknowledged that affinity purifications of eukaryotic proteasomes usually co-isolated other proteins, many of which can be considered as PIPs^24^,^26^,^27^. Consistently, apart from proteasomal components, our affinity purification method concomitantly purified a number of plasmodial proteins (Table S2). Functional analysis revealed that co-identified proteins are mainly involved in ribosome complex assembly, protein folding and quality control, translation, ubiquitin-proteasome pathway (UPP), parasite-host interaction, glycolysis, proteolysis, and redox homeostasis regulation (Fig. 3B). We found that many co-purified proteins have counterparts that have been validated as PIPs or reported to be co-purified with proteasomes in eukaryotes, including several UPS components, many chaperones, translation factors, ribosomal proteins, and some glycolytic enzymes^24^,^26^,^27^. This strongly suggests that these identified proteins could be potential PIPs for the *P. falciparum* 26S proteasome.

It is noteworthy that twelve proteins that have predicted roles in the UPP were identified, including plasmodial homologs of proteasome activator PA28 subunits, the proteasome assembly chaperone Rpn4/P27, ubiquitin, the shuttle factors PfRad23 and PfDsk2, ubiquitination enzymes (one E1 and three E2 enzymes), the ubiquitinylation mediator calcyclin binding protein, and two DUBs (Table 1). Among them are several proteins that have yeast or mammalian counterparts that have been validated as PIPs. For example, PA28, Rpn4/P27, Rad23, and Dsk2 are PIPs often seen in affinity-purifications of eukaryotic proteasomes^24^,^26^,^27^. Furthermore, PfUCH54 has been characterized as a homolog of UCH37, which is known as a proteasome-associated DUB in higher eukaryotes^12^,^28^. It also appeared that *in situ* crosslinking generally facilitated the recovery of most of these proteins, indicating a transient or weak interaction between these proteins and plasmodial proteasomes. Consistently, quantification of their relative abundance using the NSAF-based method revealed that most are present in a substoichiometric level compared to proteasome subunits (Fig. 3A), suggesting a dynamic nature of these proteins in association with the plasmodial proteasome.

### Characterization of a plasmodial proteasome-associated DUB

Of particular interest, an uncharacterized DUB (PFE1355c) annotated as a putative ubiquitin carboxyl-terminal hydrolase was reproducibly and abundantly co-identified with plasmodial proteasomes, highly suggesting a specific interaction between this DUB and the plasmodial proteasome (Table 1 and Fig. 3A). Sequence homology analysis indicated that this protein is homologous to human USP14 (hUSP14, in yeast, Ubp6), which is known as a major proteasome-associated DUB involved in trimming ubiquitin chains of proteasomal substrates^10^,^29^,^30^. Sequence alignment showed that similar to hUSP14/Ubp6, this plasmodial DUB contains an N-terminal UBL domain followed by a catalytic domain containing the catalytic triad (Cys119/His519/Asp558), which are essential for ubiquitin C-terminus hydrolysis^30^. Moreover, it additionally has two *Plasmodium*-specific insertions (E250-T324 and K527-K547), resulting in a larger size of the protein (~70.4 kDa) compared to hUSP14/Ubp6 (Fig. S5). For simplicity, we designated this DUB as PfUSP14.

**Figure 4:**
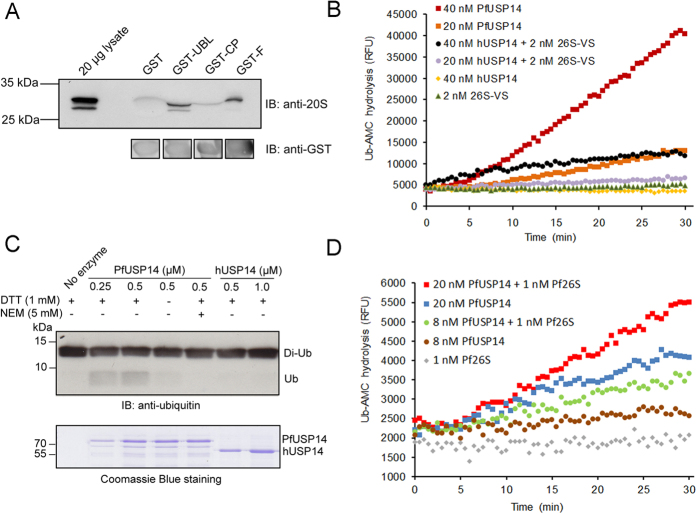
Characterizations of PfUSP14 as a proteasome-associated DUB. (**A**) Interactions of PfUSP14 with plasmodial proteasomes were examined in a GST pull-down assay using *P. falciparum* crude extracts. Equimolar amount of GST-tagged full-length PfUSP14 (GST-F), the UBL domain of PfUSP14 (GST-UBL), the catalytic part (GST-CP), or GST (control) was used for each pull-down experiment, as indicated by immunoblotting of the bait proteins using an anti-GST antibody. The pulled-down plasmodial proteasomes were resolved by 12% SDS-PAGE and detected by immunoblotting using an antibody recognizing plasmodial 20S α-subunits (Sessler *et al.*, 2012). (**B**) Robust hydrolysis of Ub-AMC by recombinant PfUSP14. Free hUSP14 was totally inactive in hydrolyzing Ub-AMC under the same experimental conditions. As a control, hUSP14 was activated by the presence of ubiquitin-vinyl sulfone (Ub-VS)-treated human proteasome (26S-VS), which lacks endogenous deubiquitinating activity. (**C**) Cleavage of Lys48-linked di-ubiquitin (Di-Ub) by recombinant PfUSP14. Omitting a reducing agent dithiothreitol (DTT) in the assay buffer or adding a thiol-blocking agent *N*-ethylmaleimide (NEM) significantly reduced PfUSP14 activity. Free hUSP14 did not cleave Di-Ub. Ubiquitin species were detected using immunoblotting with an anti-ubiquitin antibody. Coomassie blue staining was used to show the amount of the enzyme (50% input) used in each experiment. (**D**) The ubiquitin C-terminus hydrolysis activity of PfUSP14 can be enhanced by the presence of purified, Ub-VS-treated plasmodial 26S proteasomes (Pf26S). RFU, relative fluorescence unit. The results of one representative experiment are shown.

To verify the interaction between PfUSP14 and the plasmodial proteasome, we expressed and purified the recombinant full-length PfUSP14, its UBL domain, and catalytic part, which were individually fused to an N-terminal GST tag. Then binding these proteins with plasmodial proteasomes was examined by a GST-mediated pull-down assay using *P. falciparum* extracts. We found that both GST-tagged full-length PfUSP14 and the UBL domain efficiently pulled down plasmodial proteasomes, whereas the GST-tagged catalytic part and GST alone did not (Fig. 4A). This result not only corroborates the interaction of PfUSP14 with the plasmodial proteasome, but also indicates that their interaction is mediated by the UBL domain, as shown for hUSP14/Ubp6^10^,^30^.

It is known that hUSP14/Ubp6 alone hardly has ubiquitin C-terminus hydrolysis activity, whereas upon binding to the proteasome the activity can be dramatically activated^10^,^30^. To assess PfUSP14 activity, we expressed and purified hexahistidyl-tagged recombinant PfUSP14. The ubiquitin C-terminus-hydrolyzing activity of PfUSP14 was first assayed using a fluorogenic DUB substrate, ubiquitin-7-amino-4-methylcoumarin (Ub-AMC). Unexpectedly, we found that recombinant PfUSP14 exhibited a strong Ub-AMC-hydrolyzing activity. In comparison, hUSP14 alone showed no activity under the same conditions, but it can be activated by human 26S proteasomes (Fig. 4B). We next tested whether PfUSP14 acts on ubiquitin isopeptide bonds in polyubiquitin chains using a K48-linked di-ubiquitin cleavage assay. Consistently, PfUSP14 was found efficient in cleaving di-ubiquitin, resulting in the production of mono-ubiquitin, which can be clearly detected by immunoblotting analysis. PfUSP14 with oxidized or alkylated active-site cysteine failed to cleave di-ubiquitin, strongly corroborating its cysteine-dependent deubiquitinating activity. In contrast, no detectable cleavage of di-ubiquitin by hUSP14 was observed, despite higher concentrations of hUSP14 being used (Fig. 4C).

To explore whether association with proteasomes alters PfUSP14 activity, we compared PfUSP14 activity in the presence or absence of purified plasmodial 26S proteasomes devoid of endogenous deubiquitinating activity^31^. We indeed observed a slight enhancement of PfUSP14 activity in the presence of the plasmodial proteasome, indicating that binding to proteasomes also elevates PfUSP14 activity but to a lesser extent than hUSP14/Ubp6 (Fig. 4D).

### Homology modeling of PfUSP14 predicts structural differences

**Figure 5:**
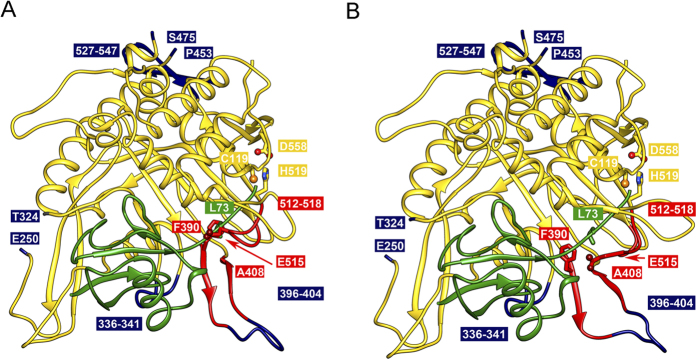
Structural model of PfUSP14. The PfUSP14 model was built based on yeast Ubp6 (1VJV). The PfUSP14-specific insertions (blue) and the putative ubiquitin (green) binding site are shown. Ubiquitin was modelled according to the structure of hUSP14 with bound ubiquitin (2AYO). The catalytic triad Cys119/His519/Asp558 is shown in a ball-and-stick model. (**A**) The conformation of the flexible loops (390–409, 512–518, red) was modelled based on the Ubp6 structure, which has been solved without bound ubiquitin. The Figure shows that the entrance of the ubiquitin C-terminal binding pocket is blocked by F390 and E515. (**B**) The flexible loops were modelled according to the structure of ubiquitin-bound hUSP14 (2AYO). The conformation changes of the loops allow the access of bound ubiquitin to the catalytic triad.

Since crystal structures of the catalytic domains of hUSP14/Ubp6 have been solved, we built a model of PfUSP14 based on the structure of the yeast ortholog Ubp6. The model revealed that the core structure of hUSP14/Ubp6 is conserved in PfUSP14. Like hUSP14/Ubp6, PfUSP14 possesses a ubiquitin-binding pocket, whereby the C-terminal tail of ubiquitin fits into a narrow channel and gets access to the aligned catalytic triad (Cys119/His519/Asp558) (Fig. 5). It has been shown that there are a couple of flexible regions undergoing conformational changes to enlarge the binding pocket upon binding ubiquitin, including the loops F390-A408 and G512-G518^30^. The corresponding loops in hUSP14/Ubp6 were notated as two blocking loops that restrict the access of the ubiquitin C-terminus to the catalytic triad and thus limit enzyme activity^30^. Furthermore, a Phe390 and a Glu515 residue mainly hinder the ubiquitin C-terminus from entering the channel, both residues are conserved in Ubp6 and PfUSP14 (Fig. 5A). The structures of hUSP14 with and without bound ubiquitin (2AYO, 2AYN) revealed the movement of these loops^30^. Consequently we built a second model according to the structure of ubiquitin-bound hUSP14 to mimic conformational changes of the regions upon binding ubiquitin in PfUSP14 (Fig. 5B).

Despite overall similarity, PfUSP14 and hUSP14/Ubp6 exhibit several local structural differences in the catalytic domain. There are a couple of *Plasmodium*-specific insertions with unknown functions. One insertion (396–404) is part of the blocking loop F390-A408. Another insertion (336–341) is part of the ubiquitin-binding pocket. The *Plasmodium*-specific insertions (250–324 and 527–547) are predicted to be positioned away from the ubiquitin-binding pocket, presumably not interfering with substrate binding of the enzyme.

As we clearly observed a strong DUB activity for free PfUSP14, we asked whether the blocking loops do not obstruct access of substrates. However, the inability of PfUSP14 to catalyze a fluorogenic substrate (Z-Arg-Leu-Arg-Gly-Gly-AMC) corresponding to the ubiquitin C-terminal peptide suggests that the entry of the ubiquitin C-terminus to the catalytic pocket is also restricted in PfUSP14 (Fig. S6), which is consistent with the predicted model. Considering the robust activity of free PfUSP14, it is thus likely that binding ubiquitin may induce conformation changes in the flexible regions, including the blocking loops, making the catalytic site more accessible for the C-terminus of ubiquitin. A similar induction mechanism by ubiquitin has been found in other USP family enzymes such as USP2^32^.

### The DUB inhibitor b-AP15 has strong antimalarial activity on intraerythrocytic *P. falciparum*

**Figure 6:**
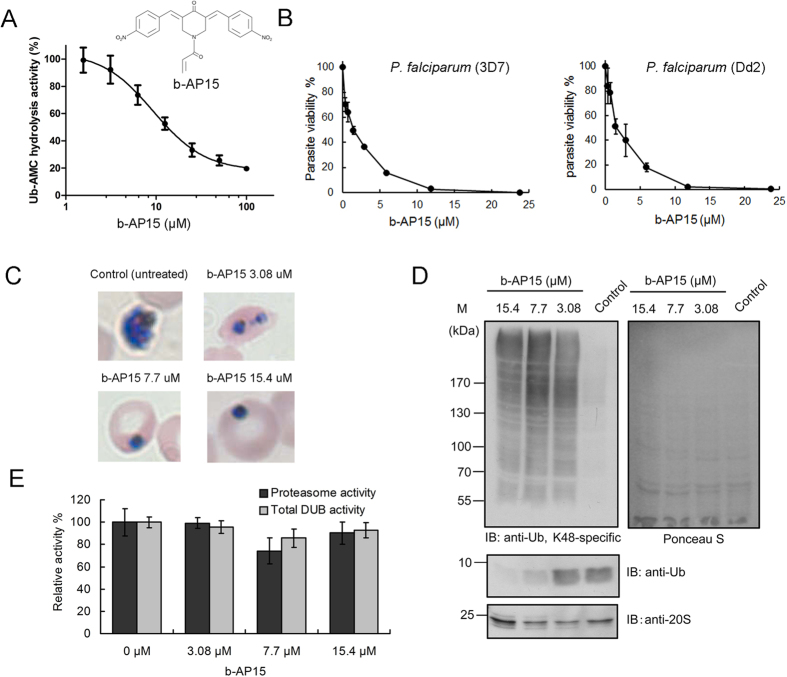
The DUB inhibitor b-AP15 inhibits PfUSP14 and exhibits antimalarial effects on intraerythrocytic *P. falciparum*. (**A**) Concentration-dependent inhibition of PfUSP14 by b-AP15. (**B**) b-AP15 potently inhibits parasite growth of chloroquine-sensitive (3D7) or chloroquine-resistant (Dd2) *P. falciparum* strains in a dose-dependent manner. **(C)** *P. falciparum* (3D7, about 38 h-old) were treated with b-AP15 of 2-fold IC_50_ (3.08 μM), 5-fold IC_50_ (7.7 μM), and 10-fold IC_50_ (15.4 μM) concentrations for 4 hours. The morphology of *P. falciparum* parasites was then analyzed in a blood smear where the parasites in red blood cells were stained with Giemsa. (**D**) Endogenous K48-linked polyubiquitinylated proteins (primary proteasomal substrates) in the b-AP15-treated parasites were resolved in 7.5% SDS-PAGE and analyzed by immunoblotting using a specific antibody recognizing K48-linked ubiquitin conjugates. Equal amounts of parasite extracts were loaded as revealed by Ponceau S staining. Free ubiquitin and the plasmodial proteasomes in the parasites were analyzed in parallel by immunoblotting using respective antibodies. (**E**) The proteasomal activity and total DUB activity in b-AP15-treated parasite were determined using a Suc-LLVY-AMC hydrolysis assay and a Ub-AMC hydrolysis assay, respectively.

We assessed the efficacy of two USP14 inhibitors, b-AP15 and IU1^33^,^34^, on PfUSP14. Interestingly, b-AP15 significantly inhibited PfUSP14 activity with an IC_50_ value of 11.3 ± 4.7 μM (Fig. 6A), whereas IU1 showed no effect on PfUSP14 up to 100 μM (data not shown). We further tested the ability of b-AP15 to inhibit the growth of intraerythrocytic *P. falciparum* parasites. It was found that treatment with b-AP15 for 68 h exerted equipotent anti-proliferative effects on both chloroquine-sensitive (3D7) and chloroquine-resistant (Dd2) strains of *P. falciparum* with IC_50_ values of 1.54 ± 0.7 μM and 1.10 ± 0.4 μM, respectively (Fig. 6B). Morphology analysis revealed that b-AP15 impeded parasite intraerythrocytic development and induced a pyknotic morphology change of the parasites after exposed to b-AP15 for a short period (4 h) in the late trophozoite stage (Fig. 6C). Additionally, we observed an accumulation of K48-linked polyubiquitinylated substrates and a depletion of the free ubiquitin pool in the treated parasites (Fig. 6D). The expression and activity of proteasomes as well as total DUB activity remained unaltered in the b-AP15-treated parasites (Fig. 6E). Thus the perturbation of proteasomal substrate turnover likely results from that the substrate deubiquitination by the proteasome-associated PfUSP14 in the parasites was affected by b-AP15.

## Discussion

In the present study we characterized the 26S proteasome complex of the malaria parasite *Plasmodium falciparum*. For the first time intrinsic ubiquitin receptor domains of the *P. falciparum* 26S proteasome could be identified. Intriguingly, only PfUIM2 was found to bind ubiquitin chains and conjugates as assessed by *in vitro* binding assays (Fig. 1). Lessons from structural studies on complexes of human ubiquitin receptors with ubiquitin may reveal structural determinants that affect the affinity of PfUIM1 and PfPru to ubiquitin. Although PfUIM1 preserves the core UIM sequence, a strictly conserved N-terminal region (^206^FGVDPS^211^ in human Rpn10) involved in the ubiquitin-binding surface of UIM1 in different species^35^ is absent in PfUIM1. It is also noticed that several key residues including Phe76, Asp78, and Phe98 in hPru, which are important for the Pru domain to bind ubiquitin^8^,^36^, have been non-conservatively substituted by Thr60, Lys61, and Asn81 in PfPru. Thus the inability of PfUIM1 and PfPru to bind ubiquitin chains might be attributed to the lack of critical residues in corresponding regions. The difference in recognition of ubiquitin chains and ubiquitinylated substrates by the plasmodial ubiquitin receptors may reveal divergent substrate recognition pathways used by the plasmodial proteasome. Our data suggest that the PfUIM2 domain in PfRpn10 serves as a primary site for direct substrate recognition by the plasmodial proteasome, whereas all PfUIM and PfPru domains may be involved in indirect substrate recognition mediated by the shuttle factors PfRad23 or PfDsk2.

The low amounts of starting materials that can be obtained from *Plasmodium* cultures, potential contaminations of host proteins, complexity of gene manipulations in *Plasmodium*, and a lack of specific antibodies against plasmodial proteasome subunits represent major challenges for the isolation of proteasomes from *Plasmodium* parasites. Our GST-UBL-based affinity purification strategy offers an uncomplicated, label-free method that can robustly isolate native *P. falciparum* 26S proteasomes directly from parasite extracts (Fig. 2B). A similar strategy has been used to purify mammalian 26S proteasomes^23^,^27^. Our established method has allowed us to unravel the componential integrity of the plasmodial 26S proteasome by showing that it contains all known proteasome subunits (Table S1). Interestingly, our data suggest a substoichiometric incorporation of PfRpn13 into plasmodial 26S proteasomes, which is reminiscent of a recent finding that only one 19S particle in a double-capped 26S proteasome contains the Rpn13 subunit^37^. Moreover, the co-purification of the 20S activator PA28 complex, which is known to attach to the 20S CP^6^, hints at that hybrid proteasomes (*i.e.* 19S-20S-PA28) and proteasome-regulating function of PA28 may exist in *P. falciparum*.

Importantly, in combination with *in situ* formaldehyde crosslinking the method was proven to be efficient in co-purifying potential PIPs of the plasmodial proteasome (Fig. 3, Table S2). In good accordance with the pattern of PIPs identified in other organisms^24^,^26^,^27^,^38^, over half of the co-purified plasmodial proteins have predicted roles in protein regulations regarding protein biosynthesis, folding, quality control, and degradation (Table S2). Of course, it is not ruled out that some proteins were captured via direct binding to the PfRad23 UBL domain or that some are polyubiquitinylated substrates transiently bound to the proteasome for degradation. However, proteasomal substrates are unlikely to contribute to the majority of identified proteins, as they are speculated to be removed by excess PfUIM2, which efficiently binds ubiquitin conjugates (Fig. 1C).

Among the co-purified proteins are a number of chaperones, notably including heat shock proteins (HSPs), T-complex protein 1 (TCP-1) subunits, and a plasmodial homolog of cell division protein 48 (CDC48) (Table S2). It has been shown that HSP70s facilitate the delivery of aggregation-prone substrates for proteasomal degradation by interacting with the 26S proteasome, and HSP90s are involved in proteasome assembly and maturation^39^,^40^,^41^. The TCP-1 complex is known as a heterooligomeric chaperone that assists in folding some cytosolic proteins^42^. A direct interaction of the TCP-1 complex with the 26S proteasome has been observed in goldfish^43^. The identification of these proteins of high abundance suggests that they might modulate protein degradation by interacting with the proteasome in *P. falciparum*. Besides, it is well acknowledged that CDC48 is a major player in the endoplasmic reticulum-associated degradation (ERAD) pathway, where CDC48, together with several cooperators, extracts and shuttles aberrant proteins from the ER to the 26S proteasome for degradation^44^. Thus our data may also indicate a linkage between the 26S proteasome and the protein quality control in ER in *P. falciparum*. Furthermore, many eukaryotic translation initiation factors (eIFs), elongation factors (eEFs) and 40S/60S ribosome subunits were abundantly co-purified (Table S2), consistent with co-purification of these proteins with proteasomes in other eukaryotes^23^,^26^,^45^. Notably, eEF1 complex has been reported to directly interact with ubiquitinylated substrates and the proteasome to mediate proteasomal degradation of co-translationally damaged proteins^9^,^46^. Therefore, the data suggest that the plasmodial proteasome may participate in cotranslational quality control and link protein synthesis and degradation pathways in *P. falciparum*.

As a validation, we identified PfUSP14 as a proteasome-associated DUB in *P. falciparum*. Most interestingly, PfUSP14 significantly differs from its human and yeast orthologs in the ubiquitin C-terminus-hydrolyzing activity. Though proteasome association elevates its activity, a considerable deubiquitinating activity was observed for unbound PfUSP14 (Fig. 4), suggesting a constant demand of the parasite on the deubiquitinating activity of PfUSP14. A timely protein turnover by the proteasome may be critical for intraerythrocytic *P. falciparum* transformations, as suggested by the observations that both ubiquitin conjugates and proteasome subunit transcription reach a maximum during late-trophozoite and schizont stages^47^,^48^. Given that proteasome deubiquitination mediated by USP14 has fundamental roles in regulating proteasomal degradation of ubiquitinylated substrates^12^,^49^, it is rational to speculate that perturbation of ubiquitin chain-trimming functions of PfUSP14 may impact intraerythrocytic parasite development and cause difficulties for parasite egress from the host cell. Strongly supporting this hypothesis, we found that the USP14 inhibitor b-AP15, which strongly inhibits recombinant PfUSP14 (Fig. 6A), exhibits potent antimalarial activity against intraerythrocytic *P. falciparum* parasites (Fig. 6B). b-AP15 has been reported to be a highly selective USP14 inhibitor that has already shown promising anti-cancer activities^33^. It should be noted that USP14 may not be the only target of b-AP15 in the parasites as b-AP15 has an order of magnitude weaker activity against the purified PfUSP14 compared to its parasite killing activity. Importantly, b-AP15 caused accumulation of proteasomal substrates and significantly reduced the free ubiquitin pool in the treated parasites, while leaving proteasomal peptidolytic activity and total DUB activity unaffected (Fig. 6D,E). These results may indicate that proteasome-mediated deubiquitination in the parasites is probably inhibited by b-AP15, which is consistent with previous reports that USP14/Ubp6 assist in the maintenance of cellular ubiquitin pools by trimming off ubiquitin from polyubiquitinylated substrates and thereby recycling it^12^,^50^. Taken together, our data suggest that targeting proteasome deubiquitinating activity, for example via inhibiting PfUSP14 activity, may represent an effective antimalarial strategy^51^,^52^.

In conclusion, this work represents a first comprehensive analysis of the *P. falciparum* 26S proteasome complex. For the first time, we successfully isolated the *P. falciparum* 26S proteasome together with potential PIPs via an affinity purification method, which may be applied in further functional and structural studies on the *P. falciparum* 26S proteasome. The putative PIPs may shed light on a profound network orchestrated by the plasmodial 26S proteasome in *P. falciparum*. Importantly, our data suggest that targeting proteasomal deubiquitinating activity in *P. falciparum* may open a new avenue for developing antimalarial strategies. We believe that further validations of other putative PIPs and characterizations of their interactions with the proteasome would greatly help not only in understanding the role of the UPS in malaria biology, but also in providing new targets for developing novel antimalarial agents.

## Material and Methods

### Gene cloning and preparation of recombinant proteins/domains

Cloning, heterologous overexpression and purification of PfUIM, PfPru, PfRad23 and PfDsk2 UBL domains as well as PfUSP14 were carried out as described in Supplementary Methods.

### Pull-down assays

To test for ubiquitin-binding abilities, 4.5 μM purified His-tagged *P. falciparum* ubiquitin receptor domains were individually mixed with 5 μg of K48- or K63-linked polyubiquitin chains ranging from one to seven (Boston Biochem) in 0.8 mL binding buffer (PBS containing 2 mg/mL BSA, 10 mM imidazole, 0.5% Triton-X100). After the mixture was incubated with Ni-NTA resin (20 μL) for 1 h at 4 °C, the beads were extensively washed with binding buffer. The bound proteins were eluted by PBS containing 500 mM imidazole. The eluted proteins were resolved by 12% sodium dodecyl sulfate- polyacrylamide gel electrophoresis (SDS-PAGE), transferred to polyvinylidene difluoride (PVDF) membrane, and probed with an anti-ubiquitin antibody (Sigma, U5379).

To test for the abilities in binding ubiquitin-conjugates, *P. falciparum* parasites (in trophozoite stage) were lysed by freezing and thawing in lysis buffer (100 mM Tris, 500 mM NaCl, pH 8.0) containing 1 mM *N*-ethylmaleimide (NEM), 100 μM MG132 and complete protease inhibitors. After centrifugation at 100,000 g, cleared supernatant was taken out and protein concentration was measured by the Bradford assay. For each pull-down experiment, 1 mg total parasite extracts and 24 μM His-tagged ubiquitin receptor domains were incubated with 25 μL Ni-NTA beads in 1.2 mL binding buffer (50 mM Tris, 150 mM NaCl, pH 8.0) for 2 h at 4 °C. After extensive wash, the bound proteins were eluted with 0.5 mL binding buffer containing 500 mM imidazole. The ubiquitin-conjugates were resolved via 7.5% SDS-PAGE, blotted on a PVDF membrane, and detected with an anti-ubiquitin antibody (Sigma, U5379).

In the UBL-binding assay, purified His-tagged *P. falciparum* ubiquitin receptor domains (18 μM) were mixed individually with 1.8 μM GST-tagged PfRad23 and PfDsk2 UBL domains together with 20 μL Ni-NTA beads in 0.8 mL binding buffer (PBS containing 2 mg/mL BSA, 10 mM imidazole, 0.5% Triton-X100). The mixture was incubated for 1 h at 4 °C, followed by an extensive wash using the binding buffer. Then the bound proteins were eluted with PBS supplemented with 500 mM imidazole. The eluted proteins were resolved via 12% SDS-PAGE and probed with anti-GST HRP conjugate (GE Healthcare).

In the *in vitro* proteasome association assay, an equimolar amount (3.5 nmol) of GST-tagged PfUSP14, catalytic part, the UBL domain, or GST (control) was individually mixed with 2 mg *P. falciparum* extracts in 1.2 mL binding buffer (50 mM Tris, 5 mM MgCl_2_, 10% glycerol, 10 mM ATP, 1 mM DTT, 1 mM Na_3_VO_4_, pH 7.4). Then the mixture was incubated with 60 μL glutathione sepharose 4B resin for 2 h at 4 °C. After an intensive wash with the binding buffer, the bound proteins were eluted with 400 μL eluting buffer (50 mM Tris, 10 mM glutathione, 5 mM DTT, pH 7.6). The eluted proteins were resolved by 12% SDS-PAGE and probed using an anti-20S subunits antibody (MCP231, Enzo life science) recognizing plasmodial proteasomes^53^. Membrane blots were probed with anti-GST antibody to assure comparable loadings of GST and GST fusion proteins.

### Harvest of *P. falciparum* and preparation of parasite extracts

To harvest *P. falciparum* parasites, intraerythrocytic *P. falciparum* (3D7) were cultivated in 24 large culturing plates (45 mL) and collected in trophozoite and early schizont stages with 8–10% parasitemia. The red blood cells were lysed in a 20-fold volume of saponin lysis buffer (7 mM K_2_HPO_4_, 1 mM NaH_2_PO_4_, 11 mM NaHCO_3_, 58 mM KCl, 56 mM NaCl, 1 mM MgCl_2_, 14 mM glucose, and 0.02% saponin, pH 7.4) for 10 min at 37 °C. After lysis, the parasite pellet was washed once with the saponin buffer followed by washing with PBS for twice. If *in situ* formaldehyde crosslinking was required, the parasites collected from two culturing plates were incubated with 10 mL PBS containing 1% formaldehyde (v/v) for 10 min at RT. Crosslinking was stopped by centrifuging the parasites at 1,500 g for 10 min immediately followed by washing with 10 mL PBS containing 0.125 M glycine^24^. After a centrifugation at 1,500 g for another 10 min, the parasite pellet was washed twice with PBS and immediately used or kept at −80 °C.

To prepare parasite extracts for proteasome purification, the parasite pellet was resuspended with an equal volume of pre-cooled proteasome purification buffer (25 mM HEPES/KOH, 5 mM MgCl_2_, 10% glycerol, 1 mM DTT and 1 mM Na_3_VO_4,_ pH 7.4) containing 10 mM ATP and complete protease inhibitors (Roche, Germany). To avoid possible disruptions of the plasmodial 26S proteasome complex, a mild protein extraction method was used to prepare *P. falciparum* cell extracts. The resuspended parasites were first passed through a needle with a diameter of 0.55 mm in a syringe (B. Braun, Germany). Then the parasites were passed eight times through a needle with the diameter of 0.4 mm within the same syringe on ice to lyse the parasites. After a 30 min centrifugation at 100,000 g at 4 °C, the cleared supernatant was taken out and subsequently passed through a 0.45-μm filter (Whatman) to remove residual cell debris. Protein concentration was determined by the Bradford assay. Typically, about 8.5 mg total proteins could be obtained from 24 large culturing plates of parasites. The cell extracts were kept on ice, protected from the light, and immediately used in the purification of plasmodial proteasomes.

To prepare parasite extracts to be used in the analysis of proteasome complexes or the PfUSP14 association assay, *P. falciparum* was lysed in a lysis buffer (50 mM Tris, 5 mM MgCl_2_, 10% glycerol, 10 mM ATP, 1 mM DTT, 1 mM Na_3_VO_4_, pH 7.4) containing complete protease inhibitors. Parasite extracts were prepared using the protein extraction method described above.

### Affinity purification of *P. falciparum* 26S proteasomes

The *P. falciparum* 26S proteasome complex was purified based on the affinity of the plasmodial proteasome to the UBL domain of PfRad23. *P. falciparum* cell extracts (~8.5 mg) were incubated with 2 mg purified GST-UBL together with 0.4 mL GSH-sepharose 4B resin for 3 h at 4 °C with constant rotation. After the mixture was poured into a 5 mL empty column, the beads were extensively washed with 10 mL pre-cooled proteasome purification buffer containing 2 mM ATP. For elution, the beads were carefully incubated with 0.5 mL proteasome purification buffer containing 2 mM ATP and 1 mg purified His-tagged PfUIM2, and incubated for 20 min at RT with occasional agitation. The eluate was collected, and the eluting step was repeated once followed by washing the beads with another 0.5 mL buffer. All fractions (1.5 mL) were combined and mixed with 0.25 mL Ni-NTA resin (Invitrogen), and incubated for 45 min with constant rotation at 4 °C to remove residual His-tagged PfUIM2. After the incubation, the mixture was collected into a clean centrifuge tube (0.8 mL, Pierce) and centrifuged briefly. The flow-through containing the purified proteasome was collected and concentrated to a final volume of approximately 100 μL. The purified proteasome samples were immediately used in the proteasomal activity measurements without freezing. The remaining samples were kept at −80 °C for further analyses. Mock purification with GST as the bait protein was carried out following the same procedure. In the PfUSP14 activation experiment, plasmodial proteasomes that are devoid of endogenous DUB activity were prepared as previously reported with modifications^31^. In this case, parasites were lysed in a buffer containing a high concentration of salt: 50 mM Tris, 5 mM MgCl_2_, 250 mM NaCl, 1 mM Na_3_VO_4_, 1 mM DTT, 10 mM ATP, 10% glycerol, pH 7.4. Additionally, during the affinity purification, plasmodial proteasomes on the beads were treated with 2 μM ubiquitin-vinyl sulfone (Ub-VS, Boston Biochem), which irreversibly inhibits thiol-based DUBs^29^.

### Multidimensional protein identification technology (MudPIT) and data processing

Proteomic analysis of purified plasmodial proteasome complexes were carried out using the MudPIT as described in Supplementary Methods. The relative abundance of identified proteins in the MS/MS analysis was quantified based on the NSAF as previously described^25^ and detailed in Supplementary Methods.

### Proteasome and DUB activity assays

Peptidolytic activities of the *P. falciparum* 26S proteasome were determined as previously described^54^. USP14 activity was measured by using either a Ub-AMC assay or a di-ubiquitin cleavage assay as previously described^34^. More details can be found in Supplementary Methods.

### Homology modelling of PfUSP14

The structure of PfUSP14 was modelled according to the crystal structure of Ubp6 (1VJV) as described in Supplementary Methods.

## Additional Information

**How to cite this article**: Wang, L. *et al.* Characterization of the 26S proteasome network in *Plasmodium falciparum*. *Sci. Rep.* **5**, 17818; doi: 10.1038/srep17818 (2015).

## Supplementary Material

Supplementary Dataset 1

## Figures and Tables

**Table 1:** Co-purified UPS components in affinity purifications of *P. falciparum* 26S proteasome.

PlasmoDB Accession No.	Descriptive name^a^	Peptides number^b^		Sequence coverage %^c^	
		F (4 trials)	N	F (4 trials)	N
Proteasome regulator and assembly chaperone					
PFI0370c	Subunit of proteasome activator complex, putative (PA28)	9/5/4/7	0	42.3/21.5/17.2/31.2	0
PFC0785c	Proteasome regulatory protein, putative (Rpn4/P27)	4/5/3/0	11	19.1/19.1/18.2/0	43.6
Ubiquitin and multiubiquitin chain-binding proteins					
PF13_0346	60S ribosomal protein L40/UBI (Ubiquitin)	3/3/2/5	2	26.6/32/19.5/32.8	14.1
PF10_0114	DNA repair protein RAD23, putative	2/2/0/2	0	5.6/5.9/0/5.6	0
PF11_0142	Ubiquitin domain containing protein (Dsk2)	4/4/4/2	0	18/18/22.2/17	0
Ubiquitination enzymes and related proteins					
PFL1245w	Ubiquitin-activating enzyme E1, putative	5/5/2/10	0	5.1/5.9/2.3/16.1	0
PFE1350c	Ubiquitin conjugating enzyme 13, putative	3/7/5/4	0	24.3/63.8/52.6/52	0
PFL0190w	Ubiquitin conjugating enzyme E2, putative	4/5/3/4	3	18.4/18.4/18.4/18.4	20.4
PF13_0301	Ubiquitin conjugating enzyme, putative	2/2/3/0	0	26.2/21.8/34.2/0	0
PFL1845c	Calcyclin binding protein, putative	2/5/5/4	0	8.3/18/21.5/27.2	0
Deubiquitinases					
PFE1355c	Ubiquitin carboxyl-terminal hydrolase, putative	34/24/26/12	104	39.7/30.2/39.8/22.5	63.5
PF11_0177	Deubiquinating/deneddylating enzyme (PfUCH54)	5/2/2/0	0	14/7.7/7.3/0	0

^a^The descriptive names of the proteins were annotated in the PlasmoDB.

^b,c^The number of peptides and the corresponding sequence coverage identified by the MS/MS analysis were given for each identified protein. Values from four independent purifications with formaldehyde crosslinking and one purification without crosslinking were listed. F, “formaldehyde,” *i.e.* with formaldehyde crosslinking; N, “normal,” *i.e.* without formaldehyde crosslinking.

## References

[b1] Phyo A. P. *et al.* Emergence of artemisinin-resistant malaria on the western border of Thailand: a longitudinal study. Lancet 379, 1960–1966 (2012). 22484134 10.1016/S0140-6736(12)60484-X PMC3525980

[b2] Wellems T. E. & Plowe C. V. Chloroquine-resistant malaria. J Infect Dis 184, 770–776 (2001). 11517439 10.1086/322858

[b3] Glickman M. H. & Ciechanover A. The ubiquitin-proteasome proteolytic pathway: destruction for the sake of construction. Physiol Rev 82, 373–428 (2002). 11917093 10.1152/physrev.00027.2001

[b4] Pickart C. M. Mechanisms underlying ubiquitination. Annu Rev Biochem 70, 503–533 (2001). 11395416 10.1146/annurev.biochem.70.1.503

[b5] Kish-Trier E. & Hill C. P. Structural biology of the proteasome. Annu Rev Biophys 42, 29–49 (2013). 23414347 10.1146/annurev-biophys-083012-130417 PMC4878838

[b6] Tomko R. J. Jr. & Hochstrasser M. Molecular architecture and assembly of the eukaryotic proteasome. Annu Rev Biochem 82, 415–445 (2013). 23495936 10.1146/annurev-biochem-060410-150257 PMC3827779

[b7] Fisher R. D. *et al.* Structure and ubiquitin binding of the ubiquitin-interacting motif. J Biol Chem 278, 28976–28984 (2003). 12750381 10.1074/jbc.M302596200

[b8] Husnjak K. *et al.* Proteasome subunit Rpn13 is a novel ubiquitin receptor. Nature 453, 481–488 (2008). 18497817 10.1038/nature06926 PMC2839886

[b9] Schmidt M., Hanna J., Elsasser S. & Finley D. Proteasome-associated proteins: regulation of a proteolytic machine. Biol Chem 386, 725–737 (2005). 16201867 10.1515/BC.2005.085

[b10] Leggett D. S. *et al.* Multiple associated proteins regulate proteasome structure and function. Mol Cell 10, 495–507 (2002). 12408819 10.1016/s1097-2765(02)00638-x

[b11] Elsasser S. & Finley D. Delivery of ubiquitinated substrates to protein-unfolding machines. Nat Cell Biol 7, 742–749 (2005). 16056265 10.1038/ncb0805-742

[b12] Lee M. J., Lee B. H., Hanna J., King R. W. & Finley D. Trimming of ubiquitin chains by proteasome-associated deubiquitinating enzymes. Mol Cell Proteomics 10, R110 003871 (2011). 20823120 10.1074/mcp.R110.003871 PMC3098602

[b13] Konstantinova I. M., Tsimokha A. S. & Mittenberg A. G. Role of proteasomes in cellular regulation. Int Rev Cell Mol Biol 267, 59–124 (2008). 18544497 10.1016/S1937-6448(08)00602-3

[b14] Navon A. & Ciechanover A. The 26 S proteasome: from basic mechanisms to drug targeting. J Biol Chem 284, 33713–33718 (2009). 19812037 10.1074/jbc.R109.018481 PMC2797140

[b15] Aminake M. N., Arndt H. D. & Pradel G. The proteasome of malaria parasites: A multi-stage drug target for chemotherapeutic intervention? Int J Parasitol Drugs Drug Resist 2, 1–10 (2012). 24533266 10.1016/j.ijpddr.2011.12.001 PMC3862440

[b16] Li H. *et al.* Validation of the proteasome as a therapeutic target in Plasmodium using an epoxyketone inhibitor with parasite-specific toxicity. Chem Biol 19, 1535–1545 (2012). 23142757 10.1016/j.chembiol.2012.09.019 PMC3529830

[b17] Czesny B., Goshu S., Cook J. L. & Williamson K. C. The proteasome inhibitor epoxomicin has potent Plasmodium falciparum gametocytocidal activity. Antimicrob Agents Chemother 53, 4080–4085 (2009). 19651911 10.1128/AAC.00088-09 PMC2764187

[b18] Lindenthal C., Weich N., Chia Y. S., Heussler V. & Klinkert M. Q. The proteasome inhibitor MLN-273 blocks exoerythrocytic and erythrocytic development of Plasmodium parasites. Parasitology 131, 37–44 (2005). 16038394 10.1017/s003118200500747x

[b19] Li H. *et al.* Identification of potent and selective non-covalent inhibitors of the Plasmodium falciparum proteasome. J Am Chem Soc 136, 13562–13565 (2014). 25226494 10.1021/ja507692y PMC4183598

[b20] Wang Q., Young P. & Walters K. J. Structure of S5a bound to monoubiquitin provides a model for polyubiquitin recognition. J Mol Biol 348, 727–739 (2005). 15826667 10.1016/j.jmb.2005.03.007

[b21] Mueller T. D. & Feigon J. Structural determinants for the binding of ubiquitin-like domains to the proteasome. EMBO J 22, 4634–4645 (2003). 12970176 10.1093/emboj/cdg467 PMC212733

[b22] Livnat-Levanon N. *et al.* Reversible 26S proteasome disassembly upon mitochondrial stress. Cell Rep 7, 1371–1380 (2014). 24857655 10.1016/j.celrep.2014.04.030

[b23] Scanlon T. C. *et al.* Isolation of human proteasomes and putative proteasome-interacting proteins using a novel affinity chromatography method. Exp Cell Res 315, 176–189 (2009). 19013454 10.1016/j.yexcr.2008.10.027

[b24] Bousquet-Dubouch M. P. *et al.* Affinity purification strategy to capture human endogenous proteasome complexes diversity and to identify proteasome-interacting proteins. Mol Cell Proteomics 8, 1150–1164 (2009). 19193609 10.1074/mcp.M800193-MCP200 PMC2689779

[b25] Paoletti A. C. *et al.* Quantitative proteomic analysis of distinct mammalian Mediator complexes using normalized spectral abundance factors. Proc Natl Acad Sci USA 103, 18928–18933 (2006). 17138671 10.1073/pnas.0606379103 PMC1672612

[b26] Guerrero C., Tagwerker C., Kaiser P. & Huang L. An integrated mass spectrometry-based proteomic approach: quantitative analysis of tandem affinity-purified *in vivo* cross-linked protein complexes (QTAX) to decipher the 26 S proteasome-interacting network. Mol Cell Proteomics 5, 366–378 (2006). 16284124 10.1074/mcp.M500303-MCP200

[b27] Besche H. C., Haas W., Gygi S. P. & Goldberg A. L. Isolation of mammalian 26S proteasomes and p97/VCP complexes using the ubiquitin-like domain from HHR23B reveals novel proteasome-associated proteins. Biochemistry 48, 2538–2549 (2009). 19182904 10.1021/bi802198q PMC3811022

[b28] Artavanis-Tsakonas K. *et al.* Identification by functional proteomics of a deubiquitinating/deNeddylating enzyme in Plasmodium falciparum. Mol Microbiol 61, 1187–1195 (2006). 16925553 10.1111/j.1365-2958.2006.05307.x PMC7168409

[b29] Borodovsky A. *et al.* A novel active site-directed probe specific for deubiquitylating enzymes reveals proteasome association of USP14. EMBO J 20, 5187–5196 (2001). 11566882 10.1093/emboj/20.18.5187 PMC125629

[b30] Hu M. *et al.* Structure and mechanisms of the proteasome-associated deubiquitinating enzyme USP14. EMBO J 24, 3747–3756 (2005). 16211010 10.1038/sj.emboj.7600832 PMC1276716

[b31] Lee B. H., Finley D. & King R. W. A High-Throughput Screening Method for Identification of Inhibitors of the Deubiquitinating Enzyme USP14. Curr Protoc Chem Biol 4, 311–330 (2012). 23788557 10.1002/9780470559277.ch120078 PMC3690187

[b32] Renatus M. *et al.* Structural basis of ubiquitin recognition by the deubiquitinating protease USP2. Structure 14, 1293–1302 (2006). 16905103 10.1016/j.str.2006.06.012 PMC7126176

[b33] D’Arcy P. *et al.* Inhibition of proteasome deubiquitinating activity as a new cancer therapy. Nat Med 17, 1636–1640 (2011). 22057347 10.1038/nm.2536

[b34] Lee B. H. *et al.* Enhancement of proteasome activity by a small-molecule inhibitor of USP14. Nature 467, 179–184 (2010). 20829789 10.1038/nature09299 PMC2939003

[b35] Zhang N. *et al.* Structure of the s5a:k48-linked diubiquitin complex and its interactions with rpn13. Mol Cell 35, 280–290 (2009). 19683493 10.1016/j.molcel.2009.06.010 PMC2748877

[b36] Schreiner P. *et al.* Ubiquitin docking at the proteasome through a novel pleckstrin-homology domain interaction. Nature 453, 548–552 (2008). 18497827 10.1038/nature06924 PMC2825158

[b37] Berko D. *et al.* Inherent asymmetry in the 26S proteasome is defined by the ubiquitin receptor RPN13. J Biol Chem 289, 5609–5618 (2014). 24429290 10.1074/jbc.M113.509380 PMC3937637

[b38] Verma R. *et al.* Proteasomal proteomics: identification of nucleotide-sensitive proteasome-interacting proteins by mass spectrometric analysis of affinity-purified proteasomes. Mol Biol Cell 11, 3425–3439 (2000). 11029046 10.1091/mbc.11.10.3425 PMC15004

[b39] Luders J., Demand J. & Hohfeld J. The ubiquitin-related BAG-1 provides a link between the molecular chaperones Hsc70/Hsp70 and the proteasome. J Biol Chem 275, 4613–4617 (2000). 10671488 10.1074/jbc.275.7.4613

[b40] Wiederkehr T., Bukau B. & Buchberger A. Protein turnover: a CHIP programmed for proteolysis. Curr Biol 12, R26–28 (2002). 11790321 10.1016/s0960-9822(01)00644-3

[b41] Imai J., Maruya M., Yashiroda H., Yahara I. & Tanaka K. The molecular chaperone Hsp90 plays a role in the assembly and maintenance of the 26S proteasome. EMBO J 22, 3557–3567 (2003). 12853471 10.1093/emboj/cdg349 PMC165619

[b42] Valpuesta J. M., Martin-Benito J., Gomez-Puertas P., Carrascosa J. L. & Willison K. R. Structure and function of a protein folding machine: the eukaryotic cytosolic chaperonin CCT. FEBS Lett 529, 11–16 (2002). 12354605 10.1016/s0014-5793(02)03180-0

[b43] Horiguchi R., Dohra H. & Tokumoto T. Comparative proteome analysis of changes in the 26S proteasome during oocyte maturation in goldfish. Proteomics 6, 4195–4202 (2006). 16791828 10.1002/pmic.200600055

[b44] Wolf D. H. & Stolz A. The Cdc48 machine in endoplasmic reticulum associated protein degradation. Biochim Biophys Acta 1823, 117–124 (2012). 21945179 10.1016/j.bbamcr.2011.09.002

[b45] Wang X. & Huang L. Identifying dynamic interactors of protein complexes by quantitative mass spectrometry. Mol Cell Proteomics 7, 46–57 (2008). 17934176 10.1074/mcp.M700261-MCP200

[b46] Gonen H., Dickman D., Schwartz A. L. & Ciechanover A. Protein synthesis elongation factor EF-1 alpha is an isopeptidase essential for ubiquitin-dependent degradation of certain proteolytic substrates. Adv Exp Med Biol 389, 209–219 (1996). 8861013 10.1007/978-1-4613-0335-0_26

[b47] Ponts N. *et al.* Unraveling the ubiquitome of the human malaria parasite. J Biol Chem 286, 40320–40330 (2011). 21930698 10.1074/jbc.M111.238790 PMC3220526

[b48] Bozdech Z. *et al.* The transcriptome of the intraerythrocytic developmental cycle of Plasmodium falciparum. PLoS Biol 1, E5 (2003). 12929205 10.1371/journal.pbio.0000005 PMC176545

[b49] Sakata E. *et al.* The catalytic activity of Ubp6 enhances maturation of the proteasomal regulatory particle. Mol Cell 42, 637–649 (2011). 21658604 10.1016/j.molcel.2011.04.021

[b50] Chernova T. A. *et al.* Pleiotropic effects of Ubp6 loss on drug sensitivities and yeast prion are due to depletion of the free ubiquitin pool. J Biol Chem 278, 52102–52115 (2003). 14559899 10.1074/jbc.M310283200

[b51] Ponder E. L. & Bogyo M. Ubiquitin-like modifiers and their deconjugating enzymes in medically important parasitic protozoa. Eukaryot Cell 6, 1943–1952 (2007). 17905920 10.1128/EC.00282-07 PMC2168404

[b52] Ponts N. *et al.* Deciphering the ubiquitin-mediated pathway in apicomplexan parasites: a potential strategy to interfere with parasite virulence. PLoS One 3, e2386 (2008). 18545708 10.1371/journal.pone.0002386 PMC2408969

[b53] Sessler N., Krug K., Nordheim A., Mordmuller B. & Macek B. Analysis of the Plasmodium falciparum proteasome using Blue Native PAGE and label-free quantitative mass spectrometry. Amino Acids 43, 1119–1129 (2012). 22821270 10.1007/s00726-012-1296-9

[b54] Lu L. *et al.* Shikonin extracted from medicinal Chinese herbs exerts anti-inflammatory effect via proteasome inhibition. Eur J Pharmacol 658, 242–247 (2011). 21392503 10.1016/j.ejphar.2011.02.043 PMC3299007

