# Supplementary material for: Characterization of the 26S proteasome network in Plasmodium falciparum
Source: Sci Rep. 2015 Dec 7;5:17818. doi: 10.1038/srep17818 (PMC4671066; doi:10.1038/srep17818)
Supplement: Supplementary Dataset 1 [file srep17818-s1.doc]

**Supplementary information**

Characterization of the 26S proteasome network in *Plasmodium falciparum*

Lihui Wang, Claire Delahunty, Karin Fritz-Wolf, Stefan Rahlfs, Judith Helena Prieto, John R. Yates and Katja Becker


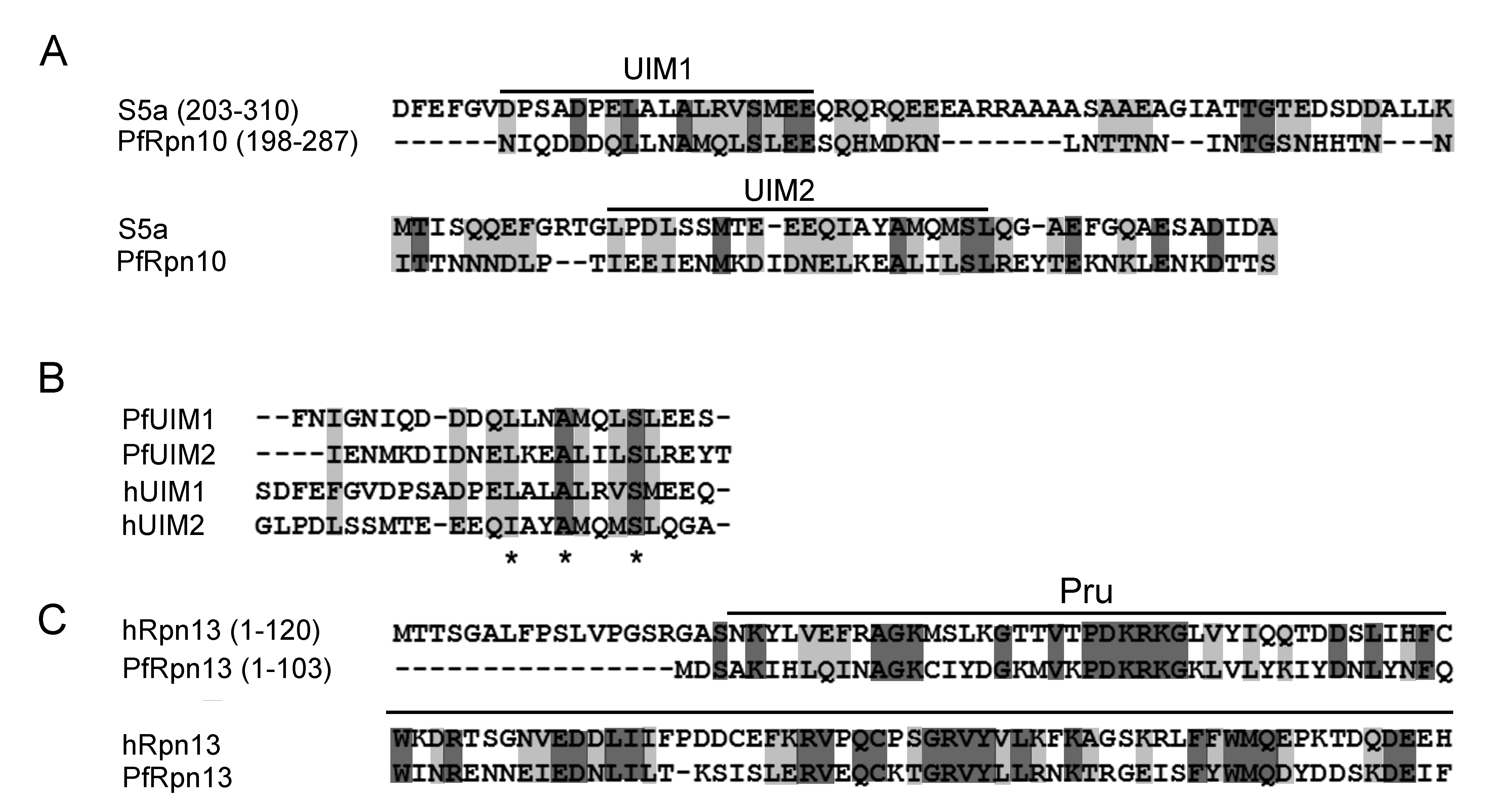


**Figure S1. *In silico* identification of putative UIM and Pru domains in *P. falciparum* 26S proteasome components. (A)** Sequence alignment of human Rpn10 (S5a_203-301_) with the corresponding region in PfRpn10 (PF08_0109). The core ubiquitin-binding patches of two human UIM domains (UIM1 and UIM2) were marked. Note that two human UIM domains are separated by about 50 amino acids. **(B)** Multiple sequence alignment of the core regions of putative PfUIM and human UIM (hUIM) domains. PfUIM domains contain the UIM consensus sequence (L/I-XX-A-XXX-S), which is essential for recognizing ubiquitin. The most conserved leucine/isoleucine, alanine and serine residues found in both PfUIM and hUIM domains were labeled with asterisks. **(C)** Sequence alignment of the N-terminus of human Rpn13 (hRpn13) and PfRpn13 (PF14_0138) revealed a putative PfPru domain. The putative PfPru domain was found to have a 57% sequence similarity with the human Pru domain. The human Pru domain is marked. In all panels, conserved residues are labeled in gray, with the intensity of the color indicating the degree of conservation of residues.


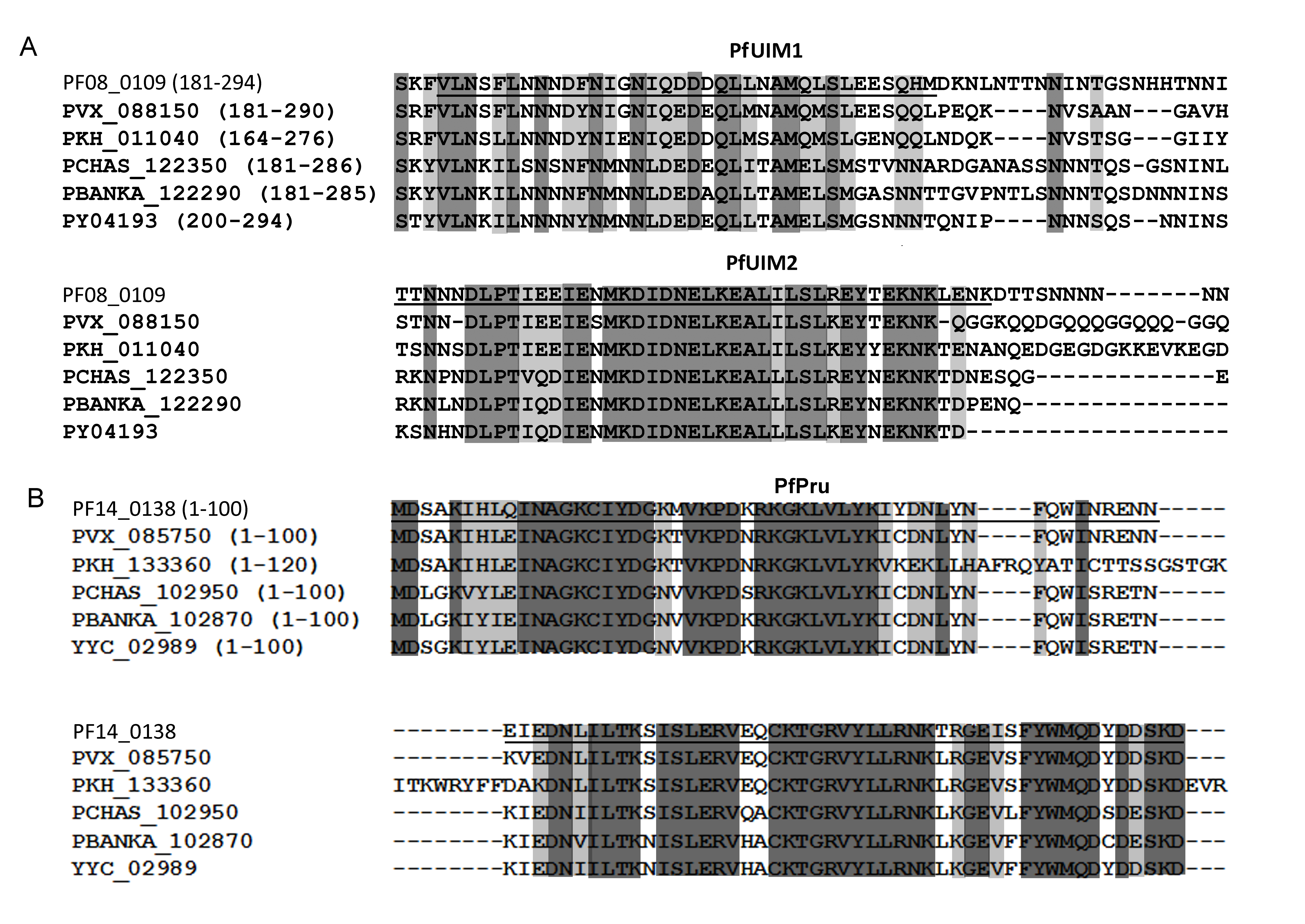


**Figure S2.** **Multiple sequence alignment of the PfUIM domains (PfRpn10_181-294_) (A) and the PfPru domain (PfRpn13_1-100_) (B) with the corresponding regions in their counterparts in other *Plasmodium* species: PVX_088150 (*P. vivax*), PKH_011040 (*P. knowlesi*), PCHAS_122350 (*P. chabaudi*), PBANKA_122290 (*P. berghei*), and PY04193 (*P. yoelii*).** The sequences of putative PfUIM and PfPru domains are underlined. The identified PfUIM domains appear to be highly conserved in *Plasmodium* species with the second UIM domain (PfUIM2) having higher degree of conservation. The identified PfPru domain is highly conserved in other *Plasmodium* species. The conserved residues are labeled in gray, with the intensity of the color indicating the degree of conservation of the residues. Sequence alignment was performed by using the program ClustalW.


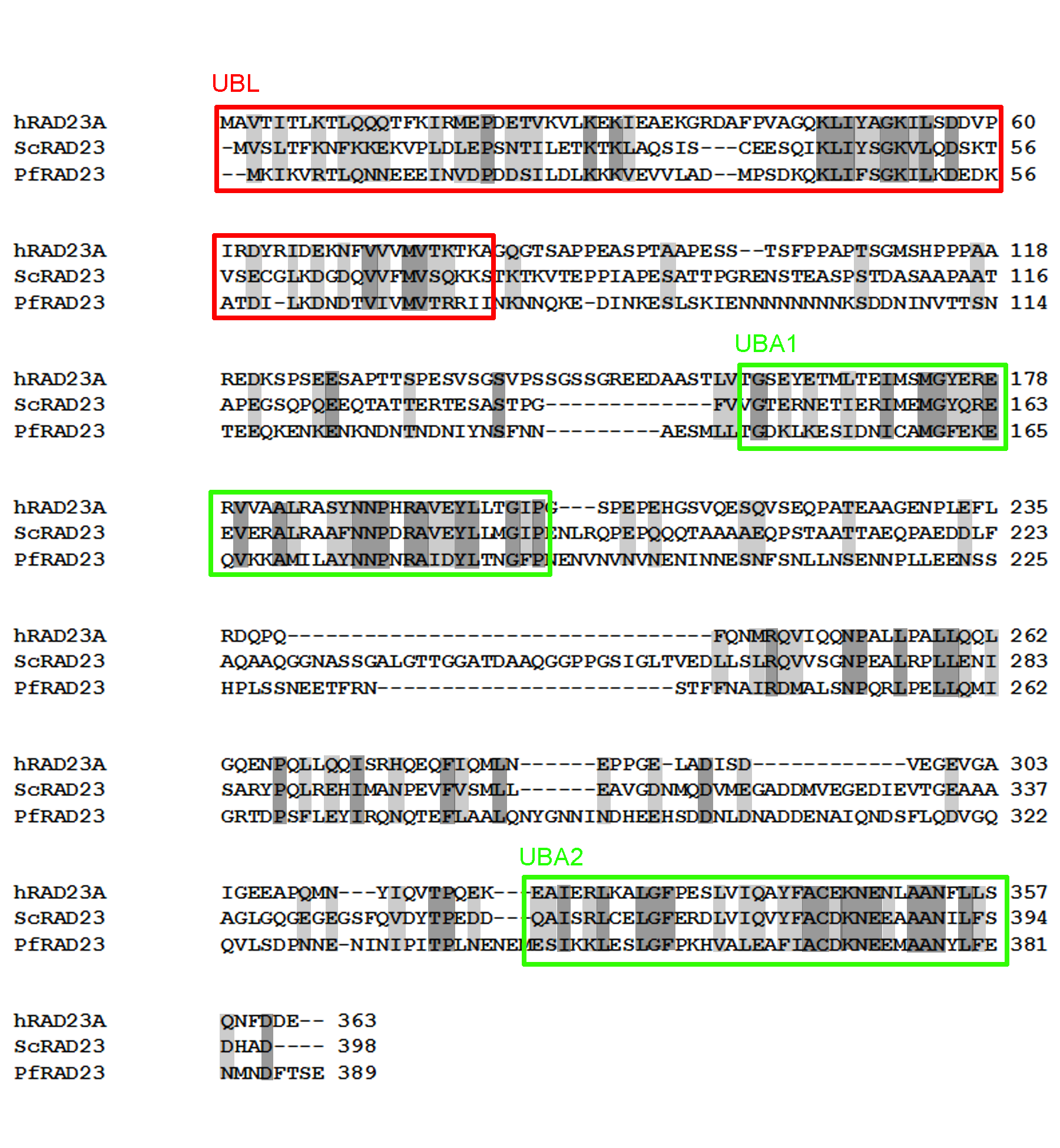


**Figure S3. Multiple sequence alignment of Rad23 homologs in human (hRad23), *Saccharomyces cerevisiae* (ScRad23), and *P. falciparum* (PfRad23, PF10_0114).** The conserved N-terminal ubiquitin-like (UBL) domain and two ubiquitin-associated (UBA) domains are boxed in red and green, respectively[^3^](#_ENREF_3). The conserved residues are labeled in gray, with the intensity of the color indicating the degree of conservation of the residues. Sequence alignment was performed by using the program ClustalW.


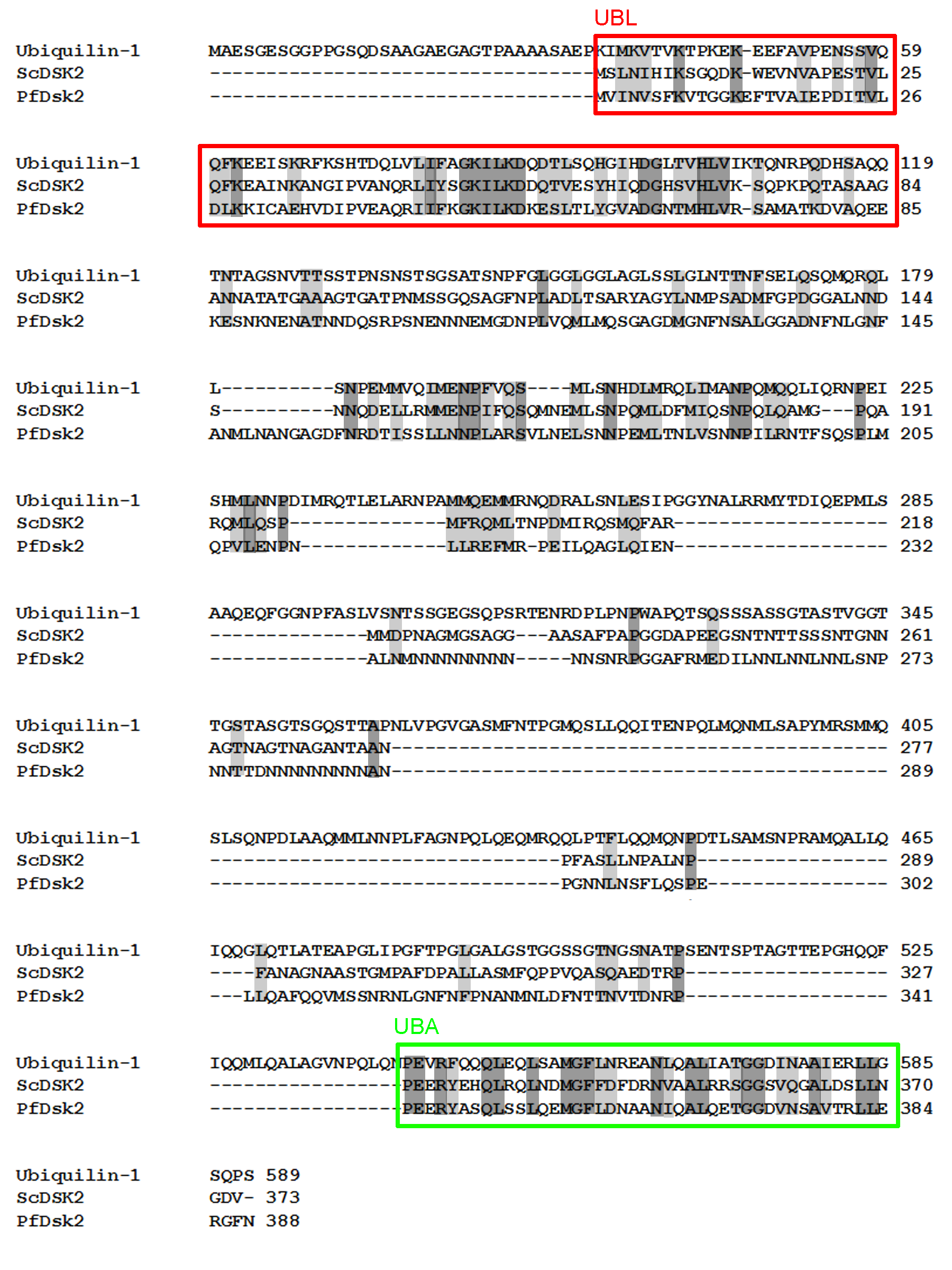


**Figure S4. Multiple sequence alignment of Dsk2 homologs in human (ubiquilin1), *Saccharomyces cerevisiae* (ScDsk2) and *P. falciparum* (PfDsk2, PF11_0142).** The conserved N-terminal UBL domain and a C-terminal UBA domain are boxed in red and green, respectively[^3^](#_ENREF_3). The conserved residues are labeled in gray, with the intensity of the color indicating the degree of conservation of the residues. Sequence alignment was performed by using the program ClustalW.


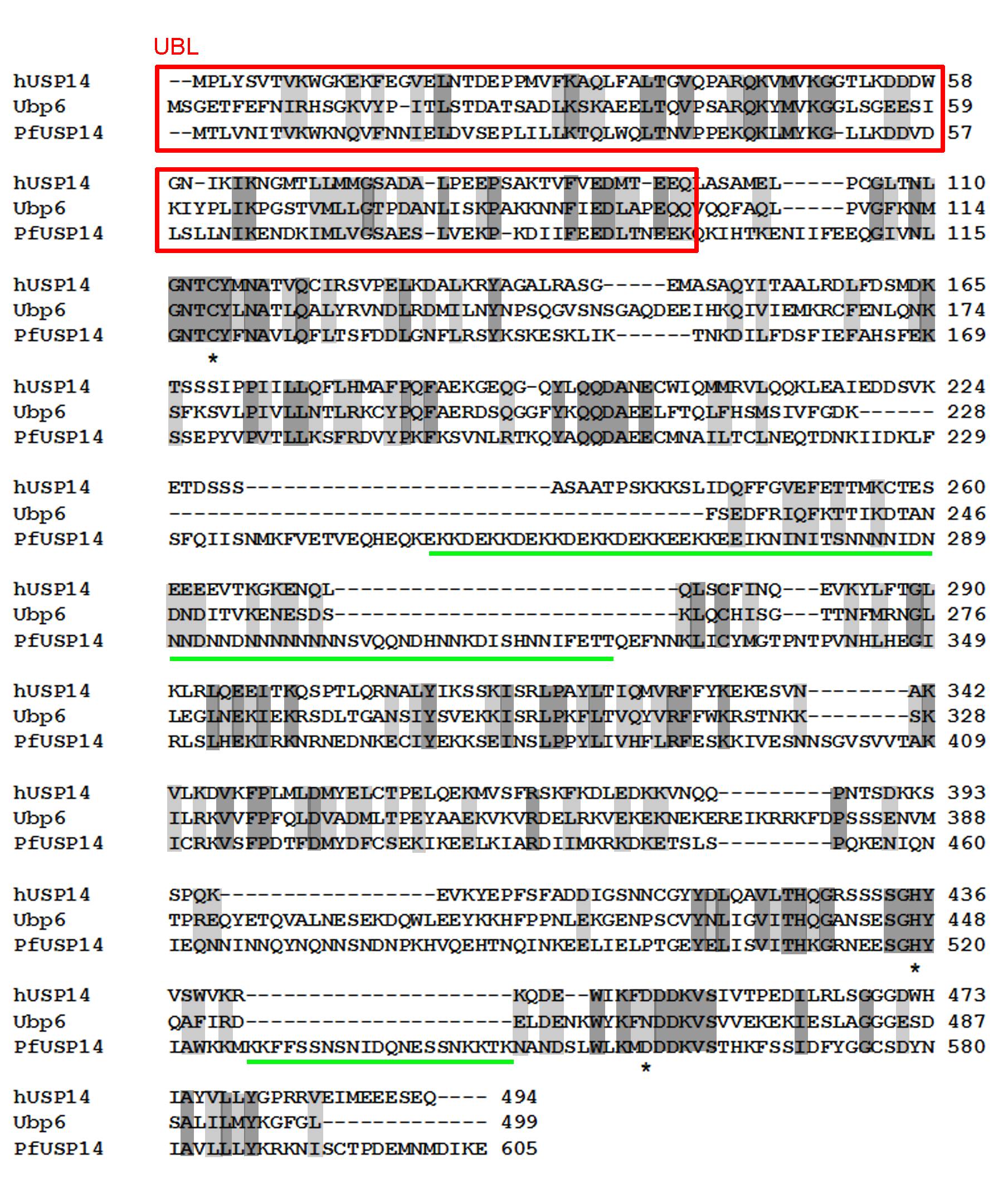


**Figure S5. Multiple sequence alignment of hUSP4, Ubp6 and PfUSP14.** The conserved N-terminal UBL domain is boxed in red. The catalytic triad (Cys119/His519/Asp558) residues are labeled with asterisks[^4^](#_ENREF_4). PfUSP14-specific insertions (E250-T324 and K527-K547) are underlined. The conserved residues are labeled in gray, with the intensity of the color indicating the degree of conservation of the residues. Sequence alignment was performed by using the program ClustalW.


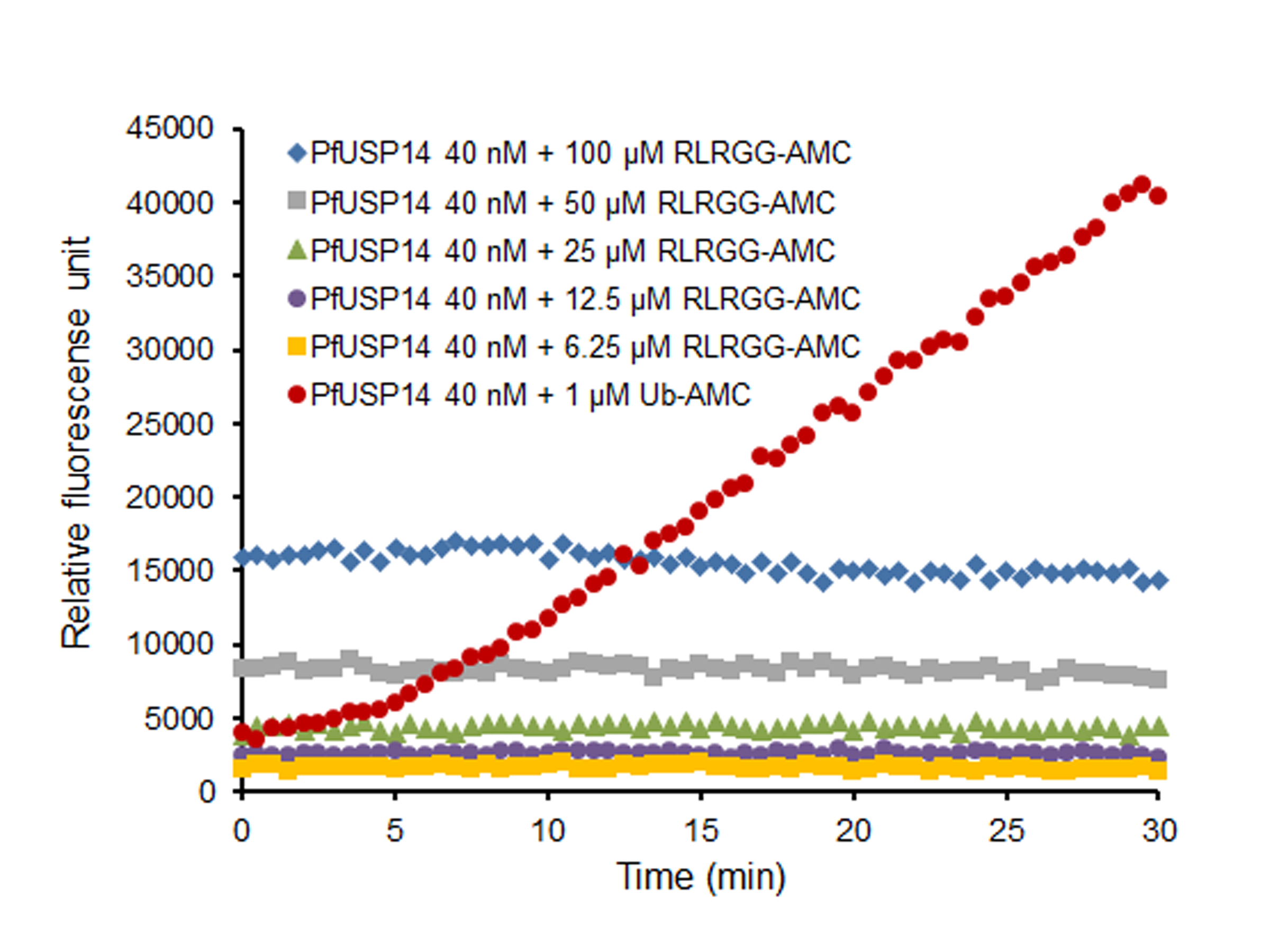


**Figure S6. PfUSP14 is unable to hydrolyze the ubiquitin C-terminal peptide Z-Arg-Leu-Arg-Gly-Gly-AMC (RLRGG-AMC).** Purified recombinant PfUSP14 (40 nM) was added individually in the assay buffer (50 mM Tris, 1 mM EDTA, 1 mM DTT, pH 7.5) containing different concentrations of RLRGG-AMC (6.25-100 μM) with a total volume of 50 μL and incubated at 37 °C. The fluorescence intensity at 460 nm (excited at 380 nm) of the released AMC was monitored for 30 min by a Tecan Infinite 200 fluorescent photometer. No catalysis of RLRGG-AMC was observed. For comparison, a robust catalysis of 1 μM ubiquitin-AMC (Ub-AMC) by PfUSP14 was observed under the same experimental condition.

**Table S1. Proteasomal components identified in the samples of affinity-purified *P. falciparum* 26S proteasomes**

T-UBL affinity chromatography fractions

| **PlasmoDB**  **Accession No.** | **Descriptive name^a^** | **Peptides number^b^** | |  | **Sequence coverage %^c^** | |
| --- | --- | --- | --- | --- | --- | --- |
|  |  | **F (4 trials)** | **N** |  | **F (4 trials)** | **N** |
|  | |  |  |  |  |  |
| **19S RP** | |  |  |  |  |  |
| **Non-ATPase subunits** | |  |  |  |  |  |
| PFB0260w | Proteasome 26S regulatory subunit, putative (Rpn1) | 55/43/28/19 | 34 |  | 38.3/32.6/24.7/25.1 | 29.5 |
| PF14_0632 | 26S proteasome regulatory subunit, putative (Rpn2) | 48/34/23/18 | 28 |  | 38.5/31.9/27.2/26.5 | 23.1 |
| MAL13P1.190 | Proteasome regulatory subunit, putative (Rpn3) | 28/19/16/6 | 5 |  | 41/34.8/40.4/19.7 | 13.7 |
| PF10_0174 | 26S proteasome regulatory subunit p55, putative (Rpn5) | 25/14/17/14 | 14 |  | 39/22.3/29.8/23.1 | 28.9 |
| PF14_0025 | Proteasome subunit, putative (Rpn6) | 22/19/15/11 | 14 |  | 27/24.2/23.9/15.3 | 19.7 |
| PF11_0303 | 26S proteasome regulatory subunit, putative (Rpn7) | 13/18/7/4 | 8 |  | 31.6/25.4/18.1/15 | 25.4 |
| PFI0630w | 26S proteasome regulatory subunit, putative (Rpn8) | 17/14/11/7 | 33 |  | 49.1/36.1/34.3/31.4 | 55.3 |
| PF10_0298 | 26S proteasome regulatory subunit, putative (Rpn9) | 16/11/5/5 | 12 |  | 34.9/30/17/16.5 | 19.6 |
| PF08_0109 | Proteasome subunit alpha type 5, putative (Rpn10) | 27/17/20/9 | 38 |  | 38.7/48.4/33.1/32.4 | 28.9 |
| MAL13P1.343 | Proteasome regulatory subunit, putative (Rpn11) | 17/15/13/13 | 58 |  | 50.8/45.7/42.8/51.1 | 71.1 |
| PFC0520w | 26S proteasome regulatory subunit S14, putative (Rpn12) | 5/3/3/2 | 2 |  | 15.8/8.9/8.9/8.9 | 15.8 |
| PF14_0138 | 26S proteasome regulatory subunit, putative (Rpn13) | 7/3/0/4 | 12 |  | 43.1/19.8/0/24.9 | 53.8 |
| **ATPase subunits** | |  |  |  |  |  |
| PF13_0063 | 26S proteasome regulatory subunit 7, putative (Rpt1) | 31/31/19/19 | 36 |  | 41.4/50.5/33.6/39.3 | 46.9 |
| PF10_0081 | 26S proteasome regulatory subunit 4, putative (Rpt2) | 42/36/37/23 | 59 |  | 62.5/60.3/53.6/52.7 | 70.8 |
| PFD0665c | 26S proteasome AAA-ATPase subunit, putative (Rpt3) | 45/29/31/15 | 86 |  | 58.7/48/59.9/37.8 | 65.1 |
| PF13_0033 | 26S proteasome regulatory subunit, putative (Rpt4) | 19/20/13/9 | 27 |  | 41.2/37.2/33.1/29.3 | 44.3 |
| PF11_0314 | 26S proteasome regulatory subunit 6a, putative (Rpt5) | 55/49/44/30 | 67 |  | 60.4/60.1/65.4/64.5 | 72 |
| PFL2345c | 26S protease regulatory subunit 8, putative (Rpt6) | 30/25/27/19 | 62 |  | 46.9/49/49/40.5 | 51.7 |
|  | |  |  |  |  |  |
| **20S CP** | |  |  |  |  |  |
| **α-subunits** | |  |  |  |  |  |
| MAL8P1.128 | Proteasome subunit alpha, putative (α1) | 16/12/8/9 | 2 |  | 53.8/46.9/28.1/35.4 | 7.3 |
| PFF0420c | Proteasome subunit alpha type 2, putative (α2) | 14/12/13/8 | 0 |  | 52.8/43.8/52.8/43.8 | 0 |
| PF13_0282 | Proteasome subunit, putative (α3) | 15/10/11/6 | 0 |  | 59.8/51.2/42.3/45.1 | 0 |
| MAL13P1.270 | Proteasome subunit, putative (α4) | 23/19/10/10 | 2 |  | 38.6/45.6/37.8/44.4 | 9.5 |
| PF07_0112 | Proteasome subunit alpha type 5, putative (α5) | 15/15/11/12 | 0 |  | 39.5/46.5/35.5/37.1 | 0 |
| PF14_0716 | Proteasome subunit alpha type 1, putative (α6) | 15/8/10/9 | 0 |  | 43.7/25.2/43.7/42.5 | 0 |
| PFC0745c | Proteasome component C8, putative (α7) | 6/6/7/3 | 0 |  | 23.8/20.6/25.8/15.9 | 0 |
| **β-subunits** | |  |  |  |  |  |
| PFI1545c | Proteasome precursor, putative (β1) | 15/11/8/3 | 0 |  | 27.3/28/26.2/21.3 | 0 |
| PF13_0156 | Proteasome subunit beta type 7 precursor, putative (β2) | 3/0/2/0 | 2 |  | 13.3/0/4.1/0 | 11.5 |
| PFA0400c | Beta3 proteasome subunit, putative (β3) | 13/10/7/5 | 0 |  | 32.4/41.7/20.6/31.4 | 0 |
| PF14_0676 | 20S proteasome beta 4 subunit, putative (β4) | 8/7/4/3 | 0 |  | 40/40/23.6/17.9 | 0 |
| PF10_0111 | 20S proteasome beta subunit, putative (β5) | 7/4/4/2 | 0 |  | 27.3/15.9/15.1/12.5 | 0 |
| PFE0915c | Proteasome subunit beta type 1, putative (β6) | 8/5/8/4 | 0 |  | 32.9/18.3/32.5/18.8 | 0 |
| MAL8P1.142 | 20S proteasome beta subunit (β7) | 30/25/14/16 | 9 |  | 44.2/44.2/38.1/45.3 | 35.8 |

^a^The descriptive names of the proteins were annotated in the PlasmoDB. The assignments of *P. falciparum* 26S proteasome subunits were cited from the “Malaria Parasite Metabolic Pathways” (http://priweb.cc.huji.ac.il/malaria/).

^b,c^The number of peptides and the corresponding sequence coverage identified by the tandem mass spectrometry (MS/MS) analysis were given for each identified protein. Values from four independent purifications with formaldehyde crosslinking and one purification without crosslinking were listed. F, “formaldehyde,” *i.e.* with formaldehyde crosslinking; N, “normal,” *i.e.* without formaldehyde crosslinking.

**Table S2. Co-identified proteins in the affinity purification of *P. falciparum* 26S proteasomes.**

| **PlasmoDB**  **Accession No.** | **Descriptive name**^a^ | **Peptides number**^b^ | |  | **Sequence coverage %**^c^ | |
| --- | --- | --- | --- | --- | --- | --- |
|  |  | **F (4 trials)** | **N** |  | **F (4 trials)** | **N** |
| **Ubiquitin-proteasome pathway** | |  |  |  |  |  |
| PF13_0346 | 60S ribosomal protein L40/UBI, putative | 3/3/2/5 | 2 |  | 26.6/32/19.5/32.8 | 14.1 |
| PFL1845c | Calcyclin binding protein, putative | 2/5/5/4 | 0 |  | 8.3/18/21.5/27.2 | 0 |
| PF11_0177 | Deubiquinating/deneddylating enzyme. | 5/2/2/0 | 0 |  | 14/7.7/7.3/0 | 0 |
| PF10_0114 | DNA repair protein RAD23, putative. | 2/2/0/2 | 0 |  | 5.6/5.9/0/5.6 | 0 |
| PFC0785c | Proteasome regulatory protein, putative (Rpn4/P27) | 4/5/3/0 | 11 |  | 19.1/19.1/18.2/0 | 43.6 |
| PFI0370c | Subunit of proteasome activator complex, putative (PA28) | 9/5/4/7 | 0 |  | 42.3/21.5/17.2/31.2 | 0 |
| PFE1355c | Ubiquitin carboxyl-terminal hydrolase, putative. | 34/24/26/12 | 104 |  | 39.7/30.2/39.8/22.5 | 63.5 |
| PFE1350c | Ubiquitin conjugating enzyme 13, putative | 3/7/5/4 | 0 |  | 24.3/63.8/52.6/52 | 0 |
| PFL0190w | Ubiquitin conjugating enzyme E2, putative | 4/5/3/4 | 3 |  | 18.4/18.4/18.4/18.4 | 20.4 |
| PF13_0301 | Ubiquitin conjugating enzyme, putative | 2/2/3/0 | 0 |  | 26.2/21.8/34.2/0 | 0 |
| PF11_0142 | Ubiquitin domain containing protein. | 4/4/4/2 | 0 |  | 18/18/22.2/17 | 0 |
| PFL1245w | Ubiquitin-activating enzyme e1, putative | 5/5/2/10 | 0 |  | 5.1/5.9/2.3/16.1 | 0 |
| **Protein folding and protein quality control** | |  |  |  |  |  |
| PFL0740c | 10 kDa chaperonin | 0/2/4/4 | 0 |  | 0/32/44.7/43.7 | 0 |
| PFF0940c | Cell division cycle protein 48 homologue, putative | 12/13/20/23 | 8 |  | 19.2/23.9/29.1/27.7 | 10.9 |
| PF07_0033 | Cg4 protein | 13/12/14/13 | 0 |  | 20.8/18.1/23.6/21 | 0 |
| PFF0430w | Chaperone, putative | 9/3/10/13 | 4 |  | 19.9/8.7/21.5/19.9 | 12 |
| PF14_0510 | Cochaperone p23 | 2/6/6/6 | 6 |  | 15.3/24.7/26.5/24.7 | 30.2 |
| PF11_0055 | Conserved protein, unknown function | 10/8/21/16 | 2 |  | 24.8/21/34/34.7 | 7.3 |
| PFL0815w | DNA-binding chaperone, putative | 6/4/5/3 | 0 |  | 8.7/5.2/8.2/5.5 | 0 |
| PF14_0700 | DnaJ protein, putative | 8/11/6/4 | 2 |  | 22.7/21.4/18.1/12.3 | 8.1 |
| PFL1070c | Endoplasmin homolog precursor, putative | 24/33/38/34 | 11 |  | 31.2/48.7/48.2/40.7 | 12.7 |
| PF10_0153 | Heat shock protein 60 | 3/2/21/16 | 3 |  | 9.3/4.3/46.9/33.3 | 8.8 |
| PF11_0351 | Heat shock protein 70 | 17/16/44/32 | 5 |  | 30.6/26.5/49.6/41.9 | 11.9 |
| PF11_0188 | Heat shock protein 90, putative | 5/3/9/7 | 3 |  | 10.1/9.6/17.5/12.5 | 4.6 |
| PF14_0359 | HSP40, subfamily A, putative | 14/4/9/9 | 11 |  | 35.1/18.2/30.7/28.1 | 26.9 |
| PFE1370w | Hsp70 interacting protein, putative | 5/2/7/7 | 0 |  | 11.6/7/26/19 | 0 |
| PF14_0324 | Hsp70/Hsp90 organizing protein, putative | 9/14/16/7 | 4 |  | 20.7/30.5/28.9/15.4 | 15.2 |
| PFF1050w | Nascent polypeptide associated complex α chain, putative | 11/8/4/5 | 0 |  | 56.5/47.8/37.5/48.9 | 0 |
| PF11_0164 | Peptidyl-prolyl cis-trans isomerase, cyclophilin | 6/4/8/7 | 4 |  | 35.9/19/51.8/46.2 | 32.3 |
| MAL8P1.17 | Protein disulfide isomerase | 17/21/25/25 | 5 |  | 37.1/42.4/50.7/49.9 | 20.1 |
| PFC0900w | T-complex protein 1 epsilon subunit, putative | 3/5/3/5 | 5 |  | 12.1/16.8/9/12.3 | 13.5 |
| PFL1425w | T-complex protein 1, gamma subunit, putative | 12/11/9/12 | 4 |  | 27.7/28.4/20.5/22.1 | 12.7 |
| PFC0285c | T-complex protein beta subunit, putative | 5/16/4/12 | 6 |  | 15.2/29.1/13.3/21.4 | 16.5 |
| PFC0350c | TCP-1/cpn60 chaperonin family, putative | 2/4/6/9 | 2 |  | 8.7/13.9/19.5/23.2 | 3.5 |
| PF11_0331 | TCP-1/cpn60 chaperonin family, putative | 6/6/6/6 | 2 |  | 17.8/17.5/17.1/16.7 | 4.8 |
| MAL13P1.283 | TCP-1/cpn60 chaperonin family, putative | 6/8/9/11 | 2 |  | 17.2/15.1/20.8/30.2 | 6.6 |
| PFA0460c | Tubulin-specific chaperone a, putative | 5/7/7/3 | 6 |  | 23.5/39.8/39.8/21.1 | 50 |
| **Translation** | |  |  |  |  |  |
| PFF1335c | 4-methyl-5(B-hydroxyethyl)-thiazol monophosphate biosynthesis enzyme | 8/8/10/9 | 3 |  | 34.4/47.6/43.9/50.3 | 18 |
| PFB0525w | Asparagine-tRNA ligase, putative | 3/6/9/6 | 2 |  | 8.5/14.6/14.9/16.9 | 6.1 |
| PF14_0546 | Conserved *Plasmodium* protein, unknown function | 2/2/6/8 | 3 |  | 5.3/5.3/13.8/14.4 | 5.2 |
| PFC0870w | Elongation factor 1 (EF-1), putative | 7/11/4/9 | 2 |  | 46.8/60.9/26.3/67.9 | 26.3 |
| PF13_0304 | Elongation factor 1-alpha | 62/63/58/63 | 62 |  | 59.8/65.9/55.1/65.9 | 56 |
| PFI0645w | Elongation factor 1-beta | 17/13/12/11 | 6 |  | 55.8/51.1/43.1/50 | 22.1 |
| PF13_0214 | Elongation factor 1-gamma, putative | 11/13/14/14 | 3 |  | 18/24.6/23.8/20.9 | 8 |
| PF14_0486 | Elongation factor 2 | 38/37/39/33 | 16 |  | 45.3/41.7/47.4/34.6 | 16.2 |
| PFL0210c | Eukaryotic initiation factor 5a, putative | 9/6/10/5 | 2 |  | 45.3/34.2/45.3/34.2 | 7.5 |
| PF14_0104 | Eukaryotic translation initiation factor 2 gamma subunit, putative | 10/10/6/7 | 0 |  | 26.2/23.4/18.3/22.2 | 0 |
| MAL7P1.81 | Eukaryotic translation initiation factor 3 37.28 kDa subunit, putative | 9/8/10/13 | 7 |  | 29.1/24.8/33/45.6 | 27.8 |
| PFL0625c | Eukaryotic translation initiation factor 3 subunit 10, putative | 10/15/7/7 | 0 |  | 10.8/14.7/9.7/9.8 | 0 |
| PF10_0077 | Eukaryotic translation initiation factor 3 subunit 7, putative | 5/2/9/8 | 0 |  | 11.6/6.6/23.4/15.7 | 0 |
| PFL0310c | Eukaryotic translation initiation factor 3 subunit 8, putative | 3/6/9/10 | 0 |  | 5.3/11.2/13.8/14.5 | 0 |
| PFF0590c | Eukaryotic translation initiation factor 3 subunit L | 6/0/4/3 | 0 |  | 11.6/0/7.2/7.2 | 0 |
| PFE1405c | Eukaryotic translation initiation factor 3, subunit 6, putative | 2/3/3/0 | 0 |  | 6/6/6/0 | 0 |
| MAL8P1.83 | Eukaryotic translation initiation factor, putative | 4/5/7/8 | 0 |  | 24.4/22.2/33.1/42.9 | 0 |
| PF13_0257 | Glutamate--tRNA ligase, putative | 11/8/8/6 | 0 |  | 16.5/11.9/12.3/10.1 | 0 |
| PF13_0170 | Glutaminyl-tRNA synthetase, putative | 0/4/4/6 | 0 |  | 0/5.7/5.7/8 | 0 |
| PF13_0179 | Isoleucine-tRNA ligase, putative | 2/2/7/10 | 0 |  | 3.5/4.4/10.5/9.4 | 0 |
| PF13_0262 | Lysine-tRNA ligase, putative | 4/4/5/5 | 0 |  | 10.8/9.6/12.9/12 | 0 |
| PF10_0340 | Methionine-tRNA ligase, putative | 9/5/10/10 | 0 |  | 11.7/10.1/12.8/13.7 | 0 |
| PFA0480w | Phenylalanyl-tRNA synthetase, putative | 2/3/0/3 | 0 |  | 3.5/6.3/0/7.3 | 0 |
| PF14_0261 | Proliferation-associated protein 2g4, putative | 10/8/5/8 | 0 |  | 26.8/21.5/12.2/18.8 | 0 |
| PF11_0245 | Translation elongation factor EF-1, subunit alpha, putative | 5/0/5/6 | 0 |  | 10.8/0/11.4/12.3 | 0 |
| PFF0345w | Translation initiation factor IF-2, putative | 0/4/8/4 | 0 |  | 0/5.8/12.5/8.9 | 0 |
| **Ribosome complex** | |  |  |  |  |  |
| PF07_0080 | 40S ribosomal protein S10, putative | 2/3/3/3 | 0 |  | 10.9/22.6/17.5/17.5 | 0 |
| PFC0775w | 40S ribosomal protein S11, putative | 4/4/4/6 | 2 |  | 16.1/16.1/17.4/31.7 | 11.2 |
| PFC0295c | 40S ribosomal protein S12, putative | 9/7/5/7 | 11 |  | 52.5/52.5/52.5/63.8 | 50.4 |
| PF13_0316 | 40S ribosomal protein S13, putative | 9/7/7/7 | 0 |  | 33.8/27.8/29.1/39.1 | 0 |
| PFE0810c | 40S ribosomal protein S14, putative | 10/12/11/13 | 14 |  | 52.3/70.9/68.9/52.3 | 73.5 |
| MAL13P1.92 | 40S ribosomal protein S15/S19, putative | 7/6/7/6 | 3 |  | 41.4/41.4/37.2/37.2 | 43.4 |
| PFC0735w | 40S ribosomal protein S15A, putative | 4/4/5/7 | 0 |  | 31.5/28.5/23.8/43.1 | 0 |
| PF08_0076 | 40S ribosomal protein S16, putative | 6/5/4/5 | 0 |  | 40.3/34/25.7/40.3 | 0 |
| PFL2055w | 40S ribosomal protein S17, putative | 9/7/7/9 | 2 |  | 56.9/43.1/49.6/56.9 | 16.8 |
| PFD1055w | 40S ribosomal protein S19, putative | 8/10/9/7 | 0 |  | 30.6/43.5/36.5/31.2 | 0 |
| PF14_0448 | 40S ribosomal protein S2, putative | 16/12/10/12 | 0 |  | 50/29/31.6/32 | 0 |
| PF11_0454 | 40S ribosomal protein S21e, putative | 5/4/4/6 | 0 |  | 50/50/50/51.2 | 0 |
| PFC0290w | 40S ribosomal protein S23, putative | 4/2/2/3 | 0 |  | 40.7/13.8/16.6/22.1 | 0 |
| PFE0975c | 40S ribosomal protein S24, putative | 3/4/4/8 | 2 |  | 24.8/28.6/24.8/41.4 | 9 |
| PF14_0205 | 40S ribosomal protein S25, putative | 6/7/6/7 | 2 |  | 34.3/35.2/33.3/42.9 | 9.5 |
| PFB0830w | 40S ribosomal protein S26e, putative | 4/4/2/4 | 0 |  | 22.4/26.2/22.4/26.2 | 0 |
| PF10_0264 | 40S ribosomal protein S2B, putative | 12/12/16/13 | 3 |  | 39.5/46/46/39.2 | 16.7 |
| PF14_0627 | 40S ribosomal protein S3, putative | 13/14/12/15 | 3 |  | 42.5/44.8/40.7/40.7 | 12.7 |
| PFC1020c | 40S ribosomal protein S3A, putative | 14/11/18/17 | 6 |  | 44.3/38.5/34.7/38.9 | 16.8 |
| PF11_0065 | 40S ribosomal protein S4, putative | 12/14/17/16 | 2 |  | 38.7/41.4/41/53.3 | 11.9 |
| PF07_0088 | 40S ribosomal protein S5, putative | 2/3/2/3 | 0 |  | 9.7/21.5/10.3/10.3 | 0 |
| PF13_0228 | 40S ribosomal protein S6, putative | 20/13/17/19 | 9 |  | 37.9/27.8/32/40.5 | 27.5 |
| PF13_0014 | 40S ribosomal protein S7, putative | 9/6/9/11 | 2 |  | 17/22.2/37.1/28.9 | 8.2 |
| PF14_0083 | 40S ribosomal protein S8e, putative | 15/13/13/10 | 2 |  | 64.7/46.8/50.5/33.9 | 13.3 |
| PFE1005w | 40S ribosomal protein S9, putative | 8/6/8/10 | 0 |  | 29.6/29.6/30.2/30.2 | 0 |
| PFC0400w | 60S acidic ribosomal protein P2, putative | 23/17/12/13 | 9 |  | 75/65.2/65.2/75 | 58 |
| PF14_0391 | 60S ribosomal protein L1, putative | 6/5/5/5 | 2 |  | 20.3/22.6/19.4/17.5 | 13.8 |
| PF14_0141 | 60S ribosomal protein L10, putative | 13/13/9/10 | 4 |  | 32/32/31.5/31.5 | 21.5 |
| PF07_0079 | 60S ribosomal protein L11a, putative | 4/3/4/4 | 3 |  | 30.6/30.6/30.6/18.5 | 13.3 |
| PFE0850c | 60S ribosomal protein L12, putative | 8/7/5/4 | 7 |  | 44.2/42.4/37.6/28.5 | 42.4 |
| PF10_0043 | 60S ribosomal protein L13, putative | 5/4/3/3 | 0 |  | 16.3/16.3/15.3/11.9 | 0 |
| PFD0770c | 60S ribosomal protein L15, putative | 5/5/7/6 | 0 |  | 26.8/23.9/23.9/23.4 | 0 |
| PF13_0268 | 60S ribosomal protein L17, putative | 7/4/5/6 | 6 |  | 23.6/15.8/20.2/20.2 | 26.6 |
| PF13_0224 | 60S ribosomal protein L18, putative | 4/6/5/8 | 8 |  | 22.3/28.8/16.8/25.5 | 27.2 |
| MAL13P1.209 | 60S ribosomal protein L18-2, putative | 3/2/7/6 | 2 |  | 20.9/12.8/21.4/21.4 | 12.8 |
| PFF0700c | 60S ribosomal protein L19, putative | 4/3/5/5 | 5 |  | 18.7/14.3/15.9/20.3 | 25.3 |
| PF14_0240 | 60S ribosomal protein L21e, putative | 5/4/5/3 | 2 |  | 24.8/26.7/27.3/19.9 | 8.1 |
| PF08_0039 | 60S ribosomal protein L22, putative | 2/4/4/3 | 0 |  | 16.5/25.9/25.9/23.7 | 0 |
| PF13_0171 | 60S ribosomal protein L23, putative | 3/3/4/3 | 0 |  | 18.7/18.7/25.9/11.5 | 0 |
| PF13_0132 | 60S ribosomal protein L23a, putative | 5/4/8/10 | 0 |  | 27.4/21.6/30/40 | 0 |
| PF13_0049 | 60S ribosomal protein L24, putative | 2/4/2/0 | 0 |  | 11.1/13.6/11.1/0 | 0 |
| PFC0535w | 60S ribosomal protein L26, putative | 7/5/6/8 | 7 |  | 24.6/24.6/24.6/31.7 | 25.4 |
| PF14_0579 | 60S ribosomal protein L27, putative | 5/3/5/4 | 7 |  | 24.7/14.4/36.3/30.8 | 24 |
| PFF0885w | 60S ribosomal protein L27a, putative | 7/6/8/10 | 10 |  | 30.4/24.3/30.4/30.4 | 33.1 |
| PF10_0272 | 60S ribosomal protein L3, putative | 13/19/15/19 | 7 |  | 26.9/33.9/30.1/33.4 | 20.5 |
| PFI0190w | 60S ribosomal protein L32, putative | 4/6/4/3 | 4 |  | 29/21.4/20.6/13 | 23.7 |
| PF07_0043 | 60S ribosomal protein L34a, putative | 4/4/2/3 | 2 |  | 20.7/20.7/10.7/15.3 | 10 |
| PF11_0260 | 60S ribosomal protein L35, putative | 3/4/3/5 | 6 |  | 21/27.4/10.5/22.6 | 15.3 |
| PF11_0312 | 60S ribosomal protein L38e, putative | 2/4/3/3 | 3 |  | 25.3/39.1/37.9/37.9 | 32.2 |
| PFE0350c | 60S ribosomal protein L4, putative | 23/19/23/18 | 10 |  | 33.6/32.4/37/33.1 | 26.8 |
| PF13_0129 | 60S ribosomal protein L6, putative | 3/3/4/3 | 0 |  | 13.2/15.8/35.8/25.8 | 0 |
| PF13_0213 | 60S ribosomal protein L6-2, putative | 8/11/11/12 | 2 |  | 41.6/42.1/41.2/48.4 | 10.4 |
| PFC0300c | 60S ribosomal protein L7, putative | 8/6/12/10 | 0 |  | 23.3/24.1/35.8/31.5 | 0 |
| PF14_0231 | 60S ribosomal protein L7-3, putative | 7/10/8/7 | 4 |  | 18.7/23.3/19.1/28.6 | 13.1 |
| PFE0845c | 60S ribosomal protein L8, putative | 5/5/3/8 | 7 |  | 15.4/20.8/12.3/22.7 | 33.1 |
| PF11_0313 | 60S ribosomal protein P0 | 20/24/21/26 | 2 |  | 52.8/70.9/63.9/57 | 10.4 |
| PF11_0106 | Apicoplast ribosomal protein L36e precursor, putative | 4/6/5/6 | 9 |  | 36.6/43.8/47.3/47.3 | 28.6 |
| **Proteolysis** | |  |  |  |  |  |
| PF14_0517 | Aminopeptidase P | 4/4/0/3 | 5 |  | 7.6/9.6/0/6.9 | 14.8 |
| PF13_0322 | Falcilysin | 2/8/10 | 0 |  | 2.8/10.5/12.4 | 0 |
| MAL13P1.56 | M1-family alanyl aminopeptidase | 15/13/26/31 | 11 |  | 21.6/18.3/27/35.1 | 16.1 |
| PF14_0327 | Methionine aminopeptidase 2 | 2/3/2/3 | 0 |  | 4.3/5.6/4.6/8.1 | 0 |
| PF14_0076 | Plasmepsin I | 8/9/8/4 | 8 |  | 24.1/21.2/20.4/15 | 29.4 |
| PF14_0075 | Plasmepsin IV | 17/13/19/15 | 26 |  | 37.6/35.4/38.1/33.2 | 40.8 |
| **Glycolysis** | |  |  |  |  |  |
| PFI0755c | 6-phosphofructokinase | 17/13/41/40 | 0 |  | 19.9/15.3/31/32 | 0 |
| PF14_0425 | Fructose-bisphosphate aldolase | 28/30/14/18 | 8 |  | 55.3/58.3/48.5/45.3 | 31.2 |
| PFF1155w | Hexokinase | 7/7/9/15 | 0 |  | 19.9/15.4/17.8/28.8 | 0 |
| PFI1105w | Phosphoglycerate kinase | 25/24/29/30 | 10 |  | 51/50.7/58.2/56 | 36.1 |
| PF11_0208 | Phosphoglycerate mutase, putative | 8/14/16/20 | 4 |  | 35.6/48.8/56.8/57.2 | 21.2 |
| PFF1300w | Pyruvate kinase | 27/22/26/38 | 12 |  | 39.5/41.9/39.3/58.1 | 33.5 |
| PF14_0378 | Triosephosphate isomerase | 5/8/11/9 | 2 |  | 14.5/36.7/44.8/39.1 | 16.1 |
| **Parasite-host interaction** | |  |  |  |  |  |
| PF11_0111 | Asparagine-rich antigen | 7/3/8/8 | 2 |  | 6.7/2.7/5.3/8.2 | 2.8 |
| PF14_0434 | Conserved Plasmodium protein, unknown function | 3/3/2/2 | 2 |  | 9.4/6.8/6.8/4.3 | 11.1 |
| PF14_0678 | Exported protein 2 | 7/11/3/2 | 2 |  | 24.7/32.4/12.9/15.3 | 4.5 |
| PFI1445w | High molecular weight rhoptry protein 2 | 7/6/17/22 | 0 |  | 8.5/7.7/16.8/16 | 0 |
| PFI0265c | High molecular weight rhoptry protein 3 | 2/3/7/0 | 0 |  | 5.9/5.1/11.4/0 | 0 |
| PFE0040c | Mature parasite-infected erythrocyte surface  antigen,erythrocyte membrane protein 2 | 7/14/64/94 | 0 |  | 5.2/9.2/31.5/38.5 | 0 |
| PF13_0197 | Merozoite surface protein 7 precursor | 4/4/9/7 | 7 |  | 18.5/11.4/24.2/18.5 | 11.4 |
| PFD0090c | Plasmodium exported protein (PHISTa), unknown function | 0/3/6/8 | 0 |  | 0/8.4/17.5/21.5 | 0 |
| PF14_0102 | Rhoptry-associated protein 1 | 6/8/4/2 | 0 |  | 11.3/13.9/9.2/5 | 0 |
| PF14_0344 | Translocon component PTEX150 | 7/9/4/0 | 0 |  | 11.6/17.5/6.2/0 | 0 |
| **Redox regulation** | |  |  |  |  |  |
| PF08_0131 | 1-cys peroxiredoxin | 2/4/0/5 | 2 |  | 12.7/14.5/0/15.9 | 19.5 |
| MAL7P1.159 | 1-cys peroxiredoxin | 4/4/11/9 | 0 |  | 24.2/32.9/46.2/33.3 | 0 |
| PF10_0268 | Peroxiredoxin (nPrx) | 4/2/8/12 | 3 |  | 10.4/6.6/18.6/28 | 12.7 |
| PF14_0368 | Thioredoxin peroxidase 1 | 7/8/14/13 | 4 |  | 28.2/42.6/46.7/43.1 | 23.6 |
| **Miscellaneous** |  |  |  |  |  |  |
| MAL8P1.69 | 14-3-3 protein, putative | 8/7/10/17 | 2 |  | 34.4/24/34.7/40.1 | 17.6 |
| PF14_0124 | Actin II | 6/8/8/7 | 16 |  | 23.9/17.6/17.6/23.9 | 29.3 |
| PF10_0016 | Acyl-CoA binding protein, isoform 2, ACBP2 | 0/2/2/2 | 0 |  | 0/30/30/30 | 0 |
| PFE1250w | Acyl-CoA synthetase, PfACS10 | 2/8/9/0 | 0 |  | 4.3/18.4/17.2/0 | 0 |
| PFE1050w | Adenosylhomocysteinase,S-adenosyl-L-homocysteine hydrolase | 10/7/10/12 | 6 |  | 24.2/19.6/22.1/24.6 | 18.8 |
| PFI0180w | Alpha tubulin 1 | 8/5/12/11 | 5 |  | 29.8/20.3/29.6/29.6 | 21.4 |
| PFC0395w | Asparagine synthetase, putative | 3/3/7/8 | 0 |  | 8.9/7.5/13.8/15.4 | 0 |
| PF14_0323 | Calmodulin | 4/2/3/3 | 2 |  | 47.7/24.8/36.2/36.2 | 35.6 |
| PFL1110c | cAMP-dependent protein kinase regulatory subunit | 2/3/8/8 | 0 |  | 5.4/8.8/12/14.5 | 0 |
| PF13_0044 | Carbamoyl phosphate synthetase | 3/3/8/9 | 0 |  | 2.3/1.9/5.6/6.5 | 0 |
| PF11_0096 | Casein kinase 2, alpha subunit | 4/3/8/11 | 0 |  | 13.4/14.3/26.6/35.2 | 0 |
| PF11_0224 | Circumsporozoite-related antigen | 6/17/20 | 0 |  | 49.4/49.4/29 | 0 |
| PFB0445c | DEAD box helicase | 9/5/2/4 | 4 |  | 27.6/13.8/8.8/13.8 | 12 |
| PF08_0095 | Dihydropteroate synthetase | 2/3/7/0 | 0 |  | 6.7/6.4/14/0 | 0 |
| MAL13P1.237 | DNA/RNA-binding protein Alba 4 | 7/6/13/15 | 2 |  | 25/22/43.5/52.2 | 7.3 |
| PF10_0063 | DNA/RNA-binding protein Alba, putative | 7/6/14/15 | 7 |  | 49.5/67.3/68.2/71 | 47.7 |
| PF08_0074 | DNA/RNA-binding protein Alba, putative | 6/3/6/8 | 2 |  | 25.8/12.1/26.6/26.2 | 8.1 |
| PF11_0257 | Ethanolamine kinase, putative | 3/2/5/8 | 0 |  | 12.8/6.1/17.3/24.8 | 0 |
| PF14_0511 | Glucose-6-phosphate dehydrogenase-6-phosphogluconolactonase | 2/5/5/0 | 0 |  | 3.3/7.6/4.2/0 | 0 |
| PF14_0187 | Glutathione S-transferase | 8/6/7/8 | 5 |  | 33.6/39.8/39.8/46.4 | 33.6 |
| MAL7P1.122 | GTP binding protein, putative | 4/6/9/9 | 0 |  | 14/25.4/38.2/37.4 | 0 |
| PF11_0183 | GTP-binding nuclear protein ran/tc4 | 12/7/11/15 | 5 |  | 50.9/41.1/46.3/63.1 | 28 |
| PF10_0325 | Haloacid dehalogenase-like hydrolase, putative | 6/8/8/9 | 0 |  | 34.4/47.9/39.6/39.6 | 0 |
| PFF0860c | Histone H2A | 0/4/8/10 | 5 |  | 0/29.5/42.4/42.4 | 24.2 |
| PFC0920w | Histone H2A variant, putative | 5/3/10/12 | 0 |  | 26.6/25.3/44.3/44.9 | 0 |
| PF11_0062 | Histone H2B | 3/3/16/21 | 0 |  | 23.9/23.9/67.5/82.1 | 0 |
| PF11_0061 | Histone H4 | 3/2/20/29 | 0 |  | 41.7/24.3/59.2/59.2 | 0 |
| PF10_0121 | Hypoxanthine phosphoribosyltransferase | 12/12/19/26 | 0 |  | 21.6/32.5/37.2/45 | 0 |
| PFI1020c | Inosine-5'-monophosphate dehydrogenase | 5/3/5/6 | 0 |  | 12/13.3/12/12 | 0 |
| PF08_0087 | Karyopherin alpha | 6/4/6/7 | 4 |  | 18.5/13.4/12.8/21.1 | 8.1 |
| PFE1195w | Karyopherin beta | 7/13/18/20 | 0 |  | 10.2/14.8/17.5/19.4 | 0 |
| PFL1420w | Macrophage migration inhibitory factor | 2/11/2/3 | 0 |  | 18.1/28.4/18.1/25.9 | 0 |
| PF14_0329 | Mitochondrial acidic protein MAM33 | 2/3/3/2 | 0 |  | 10.3/15.9/15.9/9.9 | 0 |
| PFE0585c | Myo-inositol 1-phosphate synthase, putative | 5/2/6/9 | 0 |  | 11.9/6.6/12.7/17.1 | 0 |
| PFL0185c | Nucleosome assembly protein | 7/2/2/8 | 3 |  | 24.8/10.4/6.6/25.1 | 12.4 |
| PFF0435w | Ornithine aminotransferase | 12/12/16/18 | 3 |  | 20.8/26.3/28.5/35.7 | 20.3 |
| PFL1170w | Polyadenylate-binding protein, putative | 10/19/19/12 | 3 |  | 13.5/18.3/17.6/11.9 | 7.7 |
| PFI1565w | Profilin, putative | 0/5/4/2 | 0 |  | 0/29.2/26.9/15.8 | 0 |
| PF13_0328 | Proliferating cell nuclear antigen | 0/3/3/4 | 0 |  | 0/12.4/12.4/16.4 | 0 |
| PF08_0059 | Protein kinase c inhibitor-like protein, putative | 3/3/2/2 | 0 |  | 27/17.8/17.8/17.8 | 0 |
| PFE0660c | Purine nucleoside phosphorylase | 5/9/8/11 | 0 |  | 25.3/33.9/25.3/39.6 | 0 |
| PF10_0115 | QF122 antigen | 18/16/26/23 | 13 |  | 18.8/15.4/25.5/23.8 | 13 |
| PFL2060c | Rab specific GDP dissociation inhibitor | 4/0/5/3 | 0 |  | 12.6/0/15.9/11.5 | 0 |
| PF08_0019 | Receptor for activated c kinase | 12/10/13/16 | 10 |  | 35/34.7/35/43 | 38.7 |
| PF14_0352 | Ribonucleoside-diphosphate reductase, large subunit | 6/2/4/0 | 3 |  | 10.6/5.5/6/0 | 3.1 |
| PF14_0053 | Ribonucleotide reductase small subunit | 2/6/7/0 | 3 |  | 4/17.8/14/0 | 4.6 |
| PF08_0096 | RNA helicase, putative | 2/5/5/3 | 0 |  | 3/10.8/7.9/3.2 | 0 |
| PFB0340c | Serine repeat antigen 5 | 4/2/5/4 | 0 |  | 8.5/3.4/7.9/8.9 | 0 |
| PFF1025c | SNO glutamine amidotransferase family protein | 7/8/13/18 | 0 |  | 20.3/25.6/38.5/43.2 | 0 |
| PF11_0250 | Splicing factor, putative | 2/3/2/0 | 0 |  | 13.1/27.6/13.1/0 | 0 |
| PFF0530w | Transketolase | 5/3/4/6 | 0 |  | 8.8/5.5/7.4/10.6 | 0 |
| PFE0545c | Translationally controlled tumor protein homolog, putative | 3/3/6/4 | 0 |  | 29.2/29.2/38.6/29.8 | 0 |
| PF10_0084 | Tubulin beta chain | 8/7/9/14 | 4 |  | 22.7/21.8/25.8/34.4 | 11.7 |
| PF13_0065 | Vacuolar ATP synthase subunit a | 5/10/10/8 | 0 |  | 12.4/26.7/22.1/20.3 | 0 |
| PFD0305c | Vacuolar ATP synthase subunit b | 9/7/7/14 | 0 |  | 35.2/23.3/21.9/39.1 | 0 |
| PFF0880c | Conserved Plasmodium protein, unknown function | 6/5/2/0 | 0 |  | 50.8/38.2/10.1/0 | 0 |
| PFF0835w | Conserved Plasmodium protein, unknown function | 4/5/10/10 | 5 |  | 12.8/14.6/22/26 | 15.9 |
| PFA0420w | Conserved Plasmodium protein, unknown function | 4/2/2/4 | 0 |  | 17.9/17.9/17.9/17.9 | 0 |
| PF14_0191 | Conserved Plasmodium protein, unknown function | 2/0/6/7 | 0 |  | 6.3/0/11.6/10.2 | 0 |
| PF11_0207 | Conserved Plasmodium protein, unknown function | 3/3/2/7 | 0 |  | 3.2/4.1/2.1/6.7 | 0 |
| PF08_0081 | Conserved Plasmodium protein, unknown function | 2/3/5/7 | 0 |  | 9.6/11.1/13.1/15.2 | 0 |
| MAL13P1.308 | Conserved Plasmodium protein, unknown function | 3/5/9/8 | 0 |  | 2.2/4.3/8/5.5 | 0 |
| PF14_0257 | Conserved protein, unknown function | 17/4/5/11 | 0 |  | 48/12.5/21.7/30.6 | 0 |

^a^The descriptive names of the proteins were annotated in the PlasmoDB.

^b,c^The number of peptides and the corresponding sequence coverage identified by the MS/MS analysis were given for each identified protein. Values from four independent purification trails with formaldehyde cross-linking and one purification without cross-linking were listed. F, “formaldehyde,” *i.e.* with formaldehyde cross-linking; N, “normal,” *i.e.* without formaldehyde cross-linking.

**Table S3. Identified proteins in the GST-based mock purifications of the 26S proteasome in *P. falciaprum***

| **PlasmoDB**  **Accession No.** | **Descriptive name** | **Peptides number^a^** | **Sequence coverage %** |
| --- | --- | --- | --- |
|
| PF11_0272 | 40S ribosomal protein S18, putative | 2/0 | 32.1/0 |
| PF14_0585 | 40S ribosomal protein S28e, putative | 4/0 | 32.8/0 |
| PF08_0075 | 60S ribosomal protein L13-2, putative | 2/0 | 11.2/0 |
| PF14_0230 | 60S ribosomal protein L5, putative | 2/0 | 8.8/0 |
| PF11_0043 | 60S ribosomal protein P1, putative | 3/0 | 30.5/0 |
| PFL2215w | Actin I | 2/0 | 12.8/0 |
| PF10_0086 | Adenylate kinase | 2/0 | 12/0 |
| PF11_0506 | Antigen 332, DBL-like protein | 0/3 | 0/1.7 |
| PF14_0046 | Conserved Plasmodium protein, unknown function | 0/6 | 0/28.3 |
| PFI1270w | Conserved Plasmodium protein, unknown function | 0/4 | 0/24.4 |
| MAL8P1.95 | Conserved Plasmodium protein, unknown function | 5/0 | 13.3/0 |
| PFL0280c | Conserved Plasmodium protein, unknown function | 2/0 | 12.9/0 |
| MAL13P1.233 | DNA/RNA-binding protein Alba, putative | 5/0 | 18/0 |
| MAL13P1.233 | DNA/RNA-binding protein Alba, putative | 5/0 | 18/0 |
| PF11_0098 | Endoplasmic reticulum-resident calcium binding protein | 12/2 | 46.6/7.6 |
| PF10_0155 | Enolase | 0/8 | 0/23.8 |
| PFC0271c | Glutaredoxin 1 | 3/0 | 47.7/0 |
| PF14_0598 | Glyceraldehyde-3-phosphate dehydrogenase | 9/6 | 22.8/18.4 |
| PF08_0054 | Heat shock protein 70 | 5/17 | 12.6/24.5 |
| PFI0875w | Heat shock protein 70 | 9/36 | 18.4/44.8 |
| MAL13P1.540 | Heat shock protein 70, putative | 0/2 | 0/2.6 |
| PF07_0029 | Heat shock protein 90 | 17/9 | 17.3/13.4 |
| PF14_0655 | Helicase 45 | 3/2 | 10.8/12.3 |
| PF13_0141 | L-lactate dehydrogenase | 0/5 | 0/25 |
| PFI1475w | Merozoite surface protein 1 | 16/5 | 6.1/2.7 |
| PFI0930c | Nucleosome assembly protein | 5/4 | 20.1/15.6 |
| MAL13P1.214 | Phosphoethanolamine N-methyltransferase | 0/3 | 0/12.4 |
| PF14_0077 | Plasmepsin II | 0/2 | 0/5.5 |
| PF14_0078 | Plasmepsin III,histo-aspartic protease | 0/5 | 0/12 |
| PFI0155c | Rab GTPase 7 | 3/0 | 30.1/0 |
| PFI1090w | *S*-adenosylmethionine synthetase | 0/2 | 0/7 |
| PF14_0545 | Thioredoxin, putative | 5/0 | 50/0 |
| PFD0680c | Ubiquitin carboxyl-terminal hydrolase a, putative | 2/0 | 3.3/0 |

^a^ data were collected from two independent experiments.

**Supplementary Methods**

**PCR amplification and gene cloning**

A part of the *PfRpn10* gene (PF08_0109) encoding a domain that covers both PfUIM domains was first amplified from a blood stage cDNA library of *P. falciparum* (3D7) by PCR using the primers: forward, 5’-GTTTTGAATTCATTTTTAAATAATAATG-3’; reverse, 5’-ACTTGTTGTGTCTTTATTTTCTAA TT-3’. The gene was then cloned into a pGEM-T easy vector (Promega). The respective part of the gene encoding PfUIM1 (aa 183-219), PfUIM2 (aa 241-283) and PfUIM1+2 (aa 183-283) domains were then individually amplified from the verified construct using the following primers designed to introduce BamHI and HindIII cleavage sites (underlined):

PfUIM1: forward, 5’-ATATGGATCCGTTTTGAATTCATTTTTAAATAATAATG-3’,

reverse, 5’-ATATAAGCTTCATATGCTGACTTTCTTCCAAAG-3’;

PfUIM2: forward, 5’-ATATGGATCCACAACTAATAATAATGACTTACC-3’,

reverse, 5’-ATATAAGCTTTTTATTTTCTAATTTATTCTTTTCTG-3’;

PfUIM1+2: forward, 5’-ATATGGATCCGTTTTGAATTCATTTTTAAATAATAATG-3’,

reverse, 5’-ATATAAGCTTTTTATTTTCTAATTTATTCTTTTCTG-3’.

After purification and cleavage of the PfUIM domains with respective restriction enzymes, the respective PfUIM2 and PfUIM1+2 domains were subcloned into an expression vector pQE30 (Qiagen), and the PfUIM1 domain was subcloned into an expression vector pRSET (Life Technologies). Both vectors add an N-terminal hexahistidine tag to the recombinant protein.

The N-terminal part of *PfRpn13* gene (PF14_0138) encoding the PfPru domain was directly amplified from the blood stage cDNA library of *P. falciparum* (3D7) via PCR using the primers with *BamHI* and *HindIII* cleavage sites: 5’-ATATGGATCCGATTCAGCAAAGATACATTTACA-3’ and 5’-ATATAAGCTTTTCGTCCTTCGAATCATCATAA-3’. The gene was cloned into the pQE30 vector.

The *PfRad23* (PF10_0114) and *PfDsk2* (PF11_0142) genes encoding their UBL domains were amplified from a blood stage cDNA library of *P. falciparum* (3D7) by using PCR with the following primers: PfRad23 UBL (2-83 aa): forward, 5’-ATATGGATCCAAAATAAAAGTAAG AACACTACAAAAC-3’, reverse, 5’-ATATGAATTCTTATTCCTTTTGATTATTTTTATTAAT AATTC-3’;PfDsk2 UBL (2-74 aa), 5’-ATATGGATCCGT AATAAATGTATCTTTTAAAGTTAC-3’ and 5’-ATATGAATTCTTAATTACTTCTAACTAAATG CATAGTATT-3’. After restriction enzyme digestion, then PCR products were then cloned into a GST-fusion protein expression vector pGEX-4T.

The *PfUSP14* gene (PFE1355c) was identified on chromosome 5 and amplified from a blood stage cDNA library of *P. falciparum* (3D7) via PCR using primers (forward, ATATCCATGGACATTGGTTAATATAACGGTAAAATG, reverse, ATATCTCGAGTTCCTTAA TATCCATATTCATTTCAT) that introduce NcoI and XhoI restriction sites. Cloning the complete *PfUSP14* gene was unsuccessful. Due to the presence of an internal PacI restriction site in the *PfUSP14* gene, we cloned the gene in two separate parts. The first N-terminal part was amplified using a forward primer with a BamHI restriction site (5’-ATATGGATCCACATTGGTTAATATAACGGTAAAATG-3’) and a reverse primer with a PstI restriction site (5’-ATATCTGCAGTTAATTAATTT ACTTTCTTTTGATTTA-3’); the second C-terminal part was amplified using a forward primer with a PstI restriction site (5’-ATATCTGCAGTTAATTAAAACAAATAAAGATATATTAT-3’) and a reverse primer with an EcoRI restriction site (5’-ATATCTGCAGTTAATTAATTTACTTTCTTT TGATTTA-3’). Both constructs were separately cloned into a pSK vector (Stratagene). After verification, both pSK constructs were digested with BamHI and EcoRI, and the genes were cloned into pGEX-4T vectors. To combine the two parts of *PfUSP14* in the pGEX-4T vector, both constructs were digested by PacI and EcoRI and ligated using a T4 DNA ligase (Promega). The construct containing the complete *PfSUP14* gene was verified by sequencing. A part of *PfSUP14* gene encoding an N-terminal UBL domain (2-82 aa) was amplified via PCR using the same forward primer with a BamHI restriction site and a reverse primer containing an EcoRI restriction site (underlined) (5’-ATATGAATTCTTATGGTTTCTCAACTAATGATTCTG-3’). A part of the *PfSUP14* gene encoding the catalytic domain (83-605 aa) was amplified via PCR using a forward primer with a BamHI restriction site (underlined) (5’-ATATGGATCCAAAGATATAATATTTGAAGAAGAT-3’) and the same reverse primer with an EcoRI restriction site. Both constructs were separately cloned into a pGEX-4T. The full-length *PfUSP14* gene was also amplified via PCR using the same forward primer with a BamHI restriction site and the same reverse primer with an EcoRI restriction site, and the gene was cloned into the expression vector pET28a (Novagen) with an N-terminal hexahistidyl tag.

**Heterologous overexpression and purification of recombinant proteins**

The recombinant PfUIM2, PfUIM1+2, and PfPru domains in pQE30 plasmids were overexpressed in *E. coli* M15 cells (Qiagen). For PfUIM2 and PfUIM1+2 expressions, the cells were grown in lysogeny broth (LB) medium supplemented with kanamycin (50 μg/mL) and carbenicillin (100 μg/mL) at 37 °C to optical density at 600 nm (OD600) of 0.9, and the expression was induced with 1 mM isopropyl-β-D-thiogalactopyranoside (IPTG). The cells continued to grow for 4 h and then were harvest and resuspended in a lysis buffer (50 mM sodium phosphate, 300 mM NaCl, pH 8.0) supplemented with protease inhibitors PMSF (100 μM), pepstatin (3 μM) and cystatin (80 nM). For PfPru expression, cells were grown in Terrific Broth (TB) medium supplemented with kanamycin (50 μg/mL) and carbenicillin (100 μg/mL) at 37 °C until the OD600 value reached 1.3. The cells continued to grow at RT, and when the OD600 value reached 1.6, 0.2 mM IPTG was added to induce expression. The cells were harvested after 20 h in a resuspension buffer (50 mM HEPES, 500 mM KCl, 20 mM imidazole, 10% glycerol, 1% Triton X-100, pH 8.0) supplemented with the protease inhibitors. For purification, cells were lysed by lysozyme and DNase for 30 min at 4 °C, sonicated, and centrifuged. The supernatant was applied to a Ni-NTA column (Qiagen), and His-tagged proteins were eluted with 50 mM sodium phosphate and 300 mM NaCl (pH 8.0) containing 200 mM imidazole. The proteins were further purified via gel-filtration chromatography based on a on a HiLoad 16/60 Superdex 200 prep grade column connected to an ÄKTA FPLC system (GE Healthcare) and stored in 25 mM HEPES/KOH, 5 mM MgCl_2_, 10% glycerol, pH 7.4, or 50 mM Tris, 150 mM NaCl, pH 8.0.

The recombinant PfUIM1 domain in the pRSET plasmid was expressed in *E. coli* BL21 cells (Invitrogen). The cells were grown in Terrific Broth (TB) medium supplemented with carbenicillin (100 μg/mL) at 37 °C until the OD600 value reached 0.7. The cells continued to grow at RT, and when the OD600 value reached 1.0, 0.5 mM IPTG was added to induce expression. The cells were harvest after 20 h. The recombinant PfUIM1 domain was purified following the same procedure as described for PfUIM2 and PfUIM 1+2.

The GST fusion proteins including the UBL domains of PfRad23, PfDsk2, and PfUSP14, the catalytic domain of PfUSP14, and full-length PfUSP14 in pGEX-4T plasmids were expressed in *E. coli* BL21 cells. For the expression of PfRad23 and PfDsk2 UBL domains, cells were grown in LB medium with carbenicillin (100 μg/mL) at 37 °C until the OD600 was 0.9. Expression was induced by adding 1 mM IPTG, and the cells continued to grow for 4 h. For expression of other proteins, 0.5 mM IPTG was used for induction, and cells continued to grow for 8 h. Then the cells were harvest and resuspended in PBS containing the protease inhibitors. The GST-tagged proteins were purified via a GSH Sepharose 4B column (GE Healthcare). The proteins were further purified via gel filtration and stored in 25 mM HEPES/KOH, 5 mM MgCl_2_, 10% glycerol, pH 7.4, or 50 mM Tris, 150 mM NaCl, 1 mM EDTA, pH 7.6. An untagged catalytic part of PfUSP14 was prepared from the corresponding GST-tagged protein by cleaving the N-terminal GST tag with thrombin (GE Healthcare).

The recombinant PfUSP14 in pET28a plasmid was expressed in *E. coli* BL21 cells (Invitrogen). The cells were grown in Terrific Broth (TB) medium supplemented with kanamycin (50 μg/mL) at 37 °C until the OD600 value reached 0.7. The cells continued to grow at RT, and when the OD600 value reached 1.0, 0.5 mM IPTG was added to induce expression. The cells were harvest after 20 h and suspended in 50 mM sodium phosphate, 300 mM NaCl, pH 8.0. The recombinant His-tagged PfUSP14 was purified following the same procedure as described for PfUIM2 and PfUIM 1+2. The protein was finally stored in 50 mM Tris, 150 mM NaCl, 1 mM EDTA, pH 7.6.

***P. falciparum* culture and drug treatments**

*P. falciparum* (3D7 and Dd2 strains) were cultured as previously described [^5^](#_ENREF_5). Parasites were maintained at 1 to 10% parasitemia and 3.3% hematocrit in an RPMI 1640 culture medium supplemented with A+ erythrocytes, 0.5% lipid-rich bovine serum albumin (Albumax), 9 mM (0.16%) glucose, 0.2 mM hypoxanthine, 2.1 mM L-glutamine, and 22 mg/ml gentamicin. All incubations were carried out at 37 °C in 3% O_2_, 3% CO_2_, and 94% N_2_. Synchronization of parasites in culture to ring stages was carried out via treatment with 5% (w/v) sorbitol.

To test the effects of b-AP15 and IU1 on parasite growth, twofold serial dilutions of the inhibitors were prepared in 96-well plates. Parasites were incubated with the inhibitors and chloroquine as a control at a parasitemia of 0.25% (>70% ring forms) and 1.25% hematocrit in hypoxanthine-free medium for 24 h. Then, 0.5 μCi ^3^H-hypoxanthine was added to the wells after 48 h, and the plates were incubated for a further 24 h. For determining IC_50_ values, the semi-automated microdilution technique based on ^3^H-hypoxanthine incorporation was applied[^6^](#_ENREF_6). The IC_50_ values were calculated using GraphPad Prism.

**Sample preparation for mass spectrometry**

Proteasome samples (10 μg) were diluted to 120 μL with 200 mM Tris, pH 8.5, and solid urea was added to 8 M (57 μg). Samples were reduced and alkylated by adding 6 μL of 100 mM Tris(2-carboxyethyl)phosphine (TCEP) and incubated with shaking at RT for 20 minutes, followed by addition of 6 μL of 250 mM iodoacetamide (IAA), and incubated with shaking in the dark at RT for 20 minutes. One microgram (1 μg/μL) of endoproteinase-LysC was added and the reaction was allowed to proceed for 4 hours at 37°C. Samples were diluted with 360 μL 100 mM Tris, pH 8.5, and 2 μg of trypsin (1 μg/μL) was added. Samples were incubated with shaking at 37 °C for 18 hours and digestion was halted with the addition of 25 μL of 90% formic acid. Samples were spun for 20 min at 14,000 rpm and transferred to a new Eppendorf tube.

**Multidimensional protein identification technology (MudPIT) for proteasome sample analysis**

The protein digest was pressure-loaded onto a fused silica capillary column containing 2.5 cm of Partisphere strong cation exchanger (Whatman, Clifton, NJ) followed by 2.5 cm of 10 µm Aqua C18 (Phenomenex, Ventura, CA) packed into a 250 µm inside diameter (i.d.) capillary (Polymicro Technologies, Phoenix, AZ) with a 1 µm frit. The column was washed for 60 min with buffer containing 95% water, 5% acetonitrile, and 0.1% formic acid. After washing, a 100-µm i.d. capillary with a 5-µm pulled tip packed with 15 cm 3-µm Aqua C18 material (Phenomenex, Ventura, CA) was attached via a union, and the entire split-column was placed in line with an Agilent 1100 quaternary high-performance liquid chromatography (HPLC) and analyzed using a modified 9-step separation similar to those described previously[^7^](#_ENREF_7). The buffer solutions used were 5% acetonitrile/0.1% formic acid (buffer A), 80% acetonitrile/0.1% formic acid (buffer B), and 500 mM ammonium acetate/5% acetonitrile/0.1% formic acid (buffer C). Step 1 consisted of a 90 min gradient from 0% to 100% buffer B. Steps 2-9 had the following profile: 10 min of X% buffer C, a 15 min gradient from 0% to 5% buffer B, and a 95 min gradient from 15% to 100% buffer B. The 10 min buffer C percentages were 10%, 20%, 30%, 40%, 50%, 60%, 70%, and 100%, respectively, for the 9-step analysis. As peptides were eluted from the microcapillary column, they were electrosprayed directly into an Orbitrap Velos mass spectrometer (ThermoFisher, SanJose, CA) with the application of a distal 2.4 kV spray voltage. A cycle of one full-scan mass spectrum (400-1400 m/z) followed by 15 data-dependent MS/MS spectra at a 35% normalized collision energy was repeated continuously throughout each step of the multidimensional separation. Application of mass spectrometer scan functions and HPLC solvent gradients were controlled by the XCalibur data system.

**Analysis of tandem mass spectra of proteasome samples**

Protein identifications were done with Integrated Proteomics Pipeline-IP2 (Integrated Proteomics Applications, Inc., San Diego, CA. http://www.integratedproteomics.com) using ProLuCID[^8^](#_ENREF_8) and DTASelect2.0. Spectrum raw files were extracted into ms1 and ms2 files from raw files using RawExtract 1.9.9 (http://fields.scripps.edu/downloads.php)[^11^](#_ENREF_11), and the tandem mass spectra were searched against a *Plasmodium falciparum* database (release date 01/10/12). In order to accurately estimate peptide probabilities and false discovery rates, we used a decoy database containing the reversed sequences of all the proteins appended to the target database[^12^](#_ENREF_12). Tandem mass spectra were matched to sequences using the ProLuCID algorithm. ProLuCID searches were done on an Intel Xeon cluster running under the Linux operating system. The search space included all fully and half-tryptic peptide candidates that fell within the mass tolerance window with no miscleavage constraint. Modification of +57.02146 on C (iodoacetamide) was considered to be a static modification.

The validity of peptide/spectrum matches (PSMs) was assessed in DTASelect2.0 using two SEQUEST-defined parameters[^13^](#_ENREF_13), the cross-correlation score (XCorr), and a normalized difference in cross-correlation scores (DeltaCN). The search results were grouped by charge state (+1, +2, +3, and greater than +3) and tryptic status (fully tryptic, half-tryptic), resulting in 8 distinct sub-groups. In each one of these sub-groups, the distribution of Xcorr and DeltaCN values for (a) direct and (b) decoy database PSMs was obtained; then the direct and decoy subsets were separated by a quadratic discriminant function, which was used to compute a confidence score and achieve a user-specified false discovery rate. Full separation of the direct and decoy PSM subsets is not generally possible; therefore, peptide match probabilities were calculated based on a nonparametric fit of the direct and decoy score distributions. The false discovery rate was calculated as the percentage of reverse decoy PSMs among all the PSMs that passed the confidence threshold. Identified proteins were required to have a minimum of two peptides with a mass accuracy of up to 20 ppm. Under such filtering conditions, the estimated false discovery rate was below 1% at the peptide and protein level.

**Relative quantification of protein abundance**

The relative abundance of identified proteins in the MS/MS analysis was quantified based on the normalized spectral abundance factor (NSAF)[^14^](#_ENREF_14). The NSAF of an identified protein was calculated as the number of spectral counts (SC) of the protein, divided by the protein length (L), divided by the sum of SC/L for all identified proteins in the experiment[^14^](#_ENREF_14). Statistical analysis of NSAF was carried out using Student’s *t* test as previously reported[^15^](#_ENREF_15).

**Measurement of proteasome activity**

Three peptidolytic activities of the *P. falciparum* 26S proteasome were individually determined by a fluorogenic peptide cleavage assay using three specific peptide substrates[^16^](#_ENREF_16): Suc-Leu-Leu-Val-Tyr-AMC (Suc-LLVY-AMC), Bz-Val-Gly-Arg-AMC (Bz-VGR-AMC) and Z-Leu-Leu-Glu-AMC (Z-LLE-AMC) (Enzo Life Science). In a 96-well black plate, 5 or 10 μL of purified *P. falciparum* proteasome sample was mixed with the respective substrate (100 μM) with or without 10 μM MG132 or lactacystin (Enzo life Science) in assay buffer (50 mM Tris, 5 mM MgCl_2_, 1 mM DTT, pH 7.5) with a total volume of 100 μL. The released AMC fluorescence at 460 nm (excited at 380 nm) was immediately monitored for 30 min at 37 °C by a Tecan Infinite 200 fluorescent photometer. In parallel, wells only containing the respective substrates were set as blanks. The increased fluorescence intensity (*△*F) in 30 min of blanks was subtracted from the corresponding *△*F of the respective samples. The respective proteasomal activity was calculated according to an AMC standard curve. To measure the proteasomal activity in b-AP15-treated parasites, 7.5 μg parasite extracts were used in the hydrolysis of Suc-LLVY-AMC as a substrate.

**DUB assays**

The activity of recombinant PfUSP14 and hUSP14 was determined by detecting the increase in fluorescence upon cleavage of Ub-AMC[^17^](#_ENREF_17). Purified DUBs (4 to 40 nM) were added individually in assay buffer (50 mM Tris, 1 mM EDTA, 1 mM DTT, pH 7.5) containing 1 μM Ub-AMC (Life Sensors) with a total volume of 50 μL and incubated at 37 °C. The fluorescence intensity at 460 nm (excited at 380 nm) of the released AMC was monitored for 30 min by a Tecan Infinite 200 fluorescent photometer. For testing the proteasome activation of USP14, the purified PfUSP14 or hUSP14 (Ubiquigent) were respectively mixed with purified plasmodial or human 26S proteasomes (Ubiquigent) (1 or 2 nM). After adding 1 μM Ub-AMC, the fluorescence intensity of released AMC was immediately monitored. To test the inhibitory effect of USP14 inhibitors on PfUSP14, different concentrations (0-100 μM) of b-AP15 (Merck) or IU1 (Merck) was incubated with purified PfUSP14 (40 nM) in the assay buffer, and then cleavage of Ub-AMC (1 μM) by PfUSP14 was measured. To measure the total DUB activity in b-AP15-treated parasites, 7.5 μg parasite extracts were used in the Ub-AMC hydrolysis assay.

In another experiment, the DUB activity was assessed by using K48-linked di-ubiquitin as a model substrate[^18^](#_ENREF_18). In the assay, 2 μg di-ubiquitin was incubated with PfUSP14 (250 nM and 500 nM) or hUSP14 (500 nM or 1000 nM) in 20 μL assay buffer (50 mM Tris, 1 mM EDTA, 1 mM DTT, pH 7.5) for 1 h at 37 °C. The cleavage of di-ubiquitin was monitored by 15% SDS–PAGE followed by immunoblotting using anti-ubiquitin antibody.

**Homology modelling of PfUSP14**

The structure of PfUSP14 was modelled according to the crystal structure of Ubp6 (1VJV) using the Swiss-Model automated comparative protein modelling server[^19^](#_ENREF_19). In the modelled range, the sequence similarity of PfUSP14 and 1vjv was 34%. Manuel modelling of a second model according to the structure of ubiquitin-bound hUSP14 (2AYO) was carried out with the interactive graphics program Coot[^20^](#_ENREF_20). Subsequently, the geometry minimization of the new model was performed with PHENIX[^21^](#_ENREF_21). All figures were prepared with chimera[^22^](#_ENREF_22).

**Native PAGE and in-gel proteasome activity**

*P. falciparum* extracts (90 or 120 μg) were resolved in 4% native PAGE as previously reported[^23^](#_ENREF_23). The in-gel proteasome activity assay was performed by incubating the gels for 20 min at 30 °C in an assay buffer (25 mM Tris, 10 mM MgCl_2_, 1 mM ATP, 1 mM DTT, 0.02% SDS, pH 7.4) supplemented with 0.1 mM Suc-LLVY-AMC. The in-gel proteasome activity was then visualized using a Bio-Rad Gel Doc with ultraviolet illumination.

**References**

1 Mueller, T. D. & Feigon, J. Structural determinants for the binding of ubiquitin-like domains to the proteasome. *EMBO J* **22**, 4634-4645 (2003).

2 Fisher, R. D. *et al.* Structure and ubiquitin binding of the ubiquitin-interacting motif. *J Biol Chem* **278**, 28976-28984 (2003).

3 Rao, H. & Sastry, A. Recognition of specific ubiquitin conjugates is important for the proteolytic functions of the ubiquitin-associated domain proteins Dsk2 and Rad23. *J Biol Chem* **277**, 11691-11695 (2002).

4 Hu, M. *et al.* Structure and mechanisms of the proteasome-associated deubiquitinating enzyme USP14. *EMBO J* **24**, 3747-3756 (2005).

5 Wang, L. *et al.* Protein S-nitrosylation in Plasmodium falciparum. *Antioxid Redox Signal* **20**, 2923-2935 (2014).

6 Preuss, J. *et al.* High-throughput screening for small-molecule inhibitors of plasmodium falciparum glucose-6-phosphate dehydrogenase 6-phosphogluconolactonase. *J Biomol Screen* **17**, 738-751 (2012).

7 Washburn, M. P., Wolters, D. & Yates, J. R., 3rd. Large-scale analysis of the yeast proteome by multidimensional protein identification technology. *Nat Biotechnol* **19**, 242-247 (2001).

8 Xu, T. *et al.* ProLuCID, a fast and sensitive tandem mass spectra-based protein identification program. *Mol Cell Proteomics* **5**, S174 (2006).

9 Tabb, D. L., McDonald, W. H. & Yates, J. R., 3rd. DTASelect and Contrast: tools for assembling and comparing protein identifications from shotgun proteomics. *J Proteome Res* **1**, 21-26 (2002).

10 Cociorva, D., D, L. T. & Yates, J. R. Validation of tandem mass spectrometry database search results using DTASelect. *Curr Protoc Bioinformatics* **Chapter 13**, Unit 13 14 (2007).

11 McDonald, W. H. *et al.* MS1, MS2, and SQT-three unified, compact, and easily parsed file formats for the storage of shotgun proteomic spectra and identifications. *Rapid Commun Mass Spectrom* **18**, 2162-2168 (2004).

12 Peng, J., Elias, J. E., Thoreen, C. C., Licklider, L. J. & Gygi, S. P. Evaluation of multidimensional chromatography coupled with tandem mass spectrometry (LC/LC-MS/MS) for large-scale protein analysis: the yeast proteome. *J Proteome Res* **2**, 43-50 (2003).

13 Eng, J. K., McCormack, A. L. & Yates, J. R., III. An approach to correlate tandem mass spectral data of peptides with amino acid sequences in a protein database. *J AM SOC MASS SPECTR* **5**, 976-989 (1994).

14 Paoletti, A. C. *et al.* Quantitative proteomic analysis of distinct mammalian Mediator complexes using normalized spectral abundance factors. *Proc Natl Acad Sci U S A* **103**, 18928-18933 (2006).

15 Zybailov, B. *et al.* Statistical analysis of membrane proteome expression changes in Saccharomyces cerevisiae. *J Proteome Res* **5**, 2339-2347 (2006).

16 Lu, L. *et al.* Shikonin extracted from medicinal Chinese herbs exerts anti-inflammatory effect via proteasome inhibition. *Eur J Pharmacol* **658**, 242-247 (2011).

17 Lee, B. H. *et al.* Enhancement of proteasome activity by a small-molecule inhibitor of USP14. *Nature* **467**, 179-184 (2010).

18 Lee, J. G., Baek, K., Soetandyo, N. & Ye, Y. Reversible inactivation of deubiquitinases by reactive oxygen species in vitro and in cells. *Nat Commun* **4**, 1568 (2013).

19 Arnold, K., Bordoli, L., Kopp, J. & Schwede, T. The SWISS-MODEL workspace: a web-based environment for protein structure homology modelling. *Bioinformatics* **22**, 195-201 (2006).

20 Emsley, P. & Cowtan, K. Coot: model-building tools for molecular graphics. *Acta Crystallogr D Biol Crystallogr* **60**, 2126-2132 (2004).

21 Adams, P. D. *et al.* PHENIX: a comprehensive Python-based system for macromolecular structure solution. *Acta Crystallogr D Biol Crystallogr* **66**, 213-221 (2010).

22 Pettersen, E. F. *et al.* UCSF Chimera--a visualization system for exploratory research and analysis. *J Comput Chem* **25**, 1605-1612 (2004).

23 Elsasser, S., Schmidt, M. & Finley, D. Characterization of the proteasome using native gel electrophoresis. *Methods Enzymol* **398**, 353-363 (2005).
